# A multitask clustering approach for single-cell RNA-seq analysis in Recessive Dystrophic Epidermolysis Bullosa

**DOI:** 10.1371/journal.pcbi.1006053

**Published:** 2018-04-09

**Authors:** Huanan Zhang, Catherine A. A. Lee, Zhuliu Li, John R. Garbe, Cindy R. Eide, Raphael Petegrosso, Rui Kuang, Jakub Tolar

**Affiliations:** 1 Department of Computer Science and Engineering, University of Minnesota Twin Cities, Minneapolis, Minnesota, United States of America; 2 Department of Genetics, Cell Biology and Development, University of Minnesota Twin Cities, Minneapolis, Minnesota, United States of America; 3 Minnesota Supercomputing Institute, University of Minnesota Twin Cities, Minneapolis, Minnesota, United States of America; 4 Department of Pediatrics, University of Minnesota Twin Cities, Minneapolis, Minnesota, United States of America; Toyota Technological Institute at Chicago, UNITED STATES

## Abstract

Single-cell RNA sequencing (scRNA-seq) has been widely applied to discover new cell types by detecting sub-populations in a heterogeneous group of cells. Since scRNA-seq experiments have lower read coverage/tag counts and introduce more technical biases compared to bulk RNA-seq experiments, the limited number of sampled cells combined with the experimental biases and other dataset specific variations presents a challenge to cross-dataset analysis and discovery of relevant biological variations across multiple cell populations. In this paper, we introduce a method of variance-driven multitask clustering of single-cell RNA-seq data (scVDMC) that utilizes multiple single-cell populations from biological replicates or different samples. scVDMC clusters single cells in multiple scRNA-seq experiments of similar cell types and markers but varying expression patterns such that the scRNA-seq data are better integrated than typical pooled analyses which only increase the sample size. By controlling the variance among the cell clusters within each dataset and across all the datasets, scVDMC detects cell sub-populations in each individual experiment with shared cell-type markers but varying cluster centers among all the experiments. Applied to two real scRNA-seq datasets with several replicates and one large-scale droplet-based dataset on three patient samples, scVDMC more accurately detected cell populations and known cell markers than pooled clustering and other recently proposed scRNA-seq clustering methods. In the case study applied to in-house Recessive Dystrophic Epidermolysis Bullosa (RDEB) scRNA-seq data, scVDMC revealed several new cell types and unknown markers validated by flow cytometry. MATLAB/Octave code available at https://github.com/kuanglab/scVDMC.

## Author summary

scRNA-seq enables detailed profiling of heterogeneous cell populations and can be used to reveal lineage relationships or discover new cell types. In the literature, there has been little effort directed towards developing computational methods for cross-population transcriptome analysis of multiple single-cell populations. The cross-cell-population clustering problem is different from the traditional clustering problem because single-cell populations can be collected from different patients, different samples of a tissue, or different experimental replicates. The accompanying biological and technical variation tends to dominate the signals for clustering the pooled single cells from the multiple populations. In this work, we have developed a multitask clustering method to address the cross-population clustering problem. The method simultaneously clusters each individual cell population and controls variance among the cell-type cluster centers within each cell population and across the cell populations. We demonstrate that our multitask clustering method significantly improves clustering accuracy and marker discovery in three public scRNA-seq datasets and also apply the method to an in-house Recessive Dystrophic Epidermolysis Bullosa (RDEB) dataset. Our results make it evident that multitask clustering is a promising new approach for cross-population analysis of scRNA-seq data.

## Introduction

In recent years, single-cell RNA sequencing (scRNA-seq) has emerged as the dominant method for quantifying transcriptome-wide mRNA expression in individual cells. While traditional bulk RNA-seq ignores the differences between individual cells and treats the population of cells as homogeneous, scRNA-seq identifies sub-populations of single cells and can be useful for characterizing sub-population structure, mechanisms of transcription regulation, and understanding disease progression [1] and immunology [2]. A typical scRNA-seq protocol consists of several steps: isolation of single cells and RNA, reverse transcription, amplification, library generation, and sequencing. In addition to the noise and bias that exist in bulk RNA-seq experiments, issues unique to scRNA-seq include those from biological sources, such as cell-cycle stage or cell size, as well as from technical/systematic sources, such as capture inefficiency, material degradation, sample contamination, amplification biases, GC content, and sequencing depth. These experimental biases and limitations cause uneven coverage of the entire transcriptome and result in an abundance of zero-coverage regions [3, 4].

Typically, the cost of scRNA-Seq is much higher than bulk RNA-Seq per sample, and thus, scRNA-Seq of a large patient cohort is still prohibitively costly. When a large number of single-cells from multiple samples are sequenced, more complex batch effects might be introduced. Finally, some poorly sampled cell populations might only contain very few cells for the analysis. To address all these challenges, proper integration of multiple scRNA-Seq datasets generated from different experiments is important. When multiple single-cell populations from biological replicates or related samples such as a patient cohort are analyzed to discover the common and sample-specific cell types, technical biases and irrelevant biological variance among independent samples cannot be easily identified and removed from the signal before clustering the single cells. For example, when the scRNA-seq profiles from multiple patients are pooled together for clustering, the clusters will highly overlap with the division of the single cells by the sample origins rather than similar types such as pathogenic cells vs normal cells.

**Fig 1:**
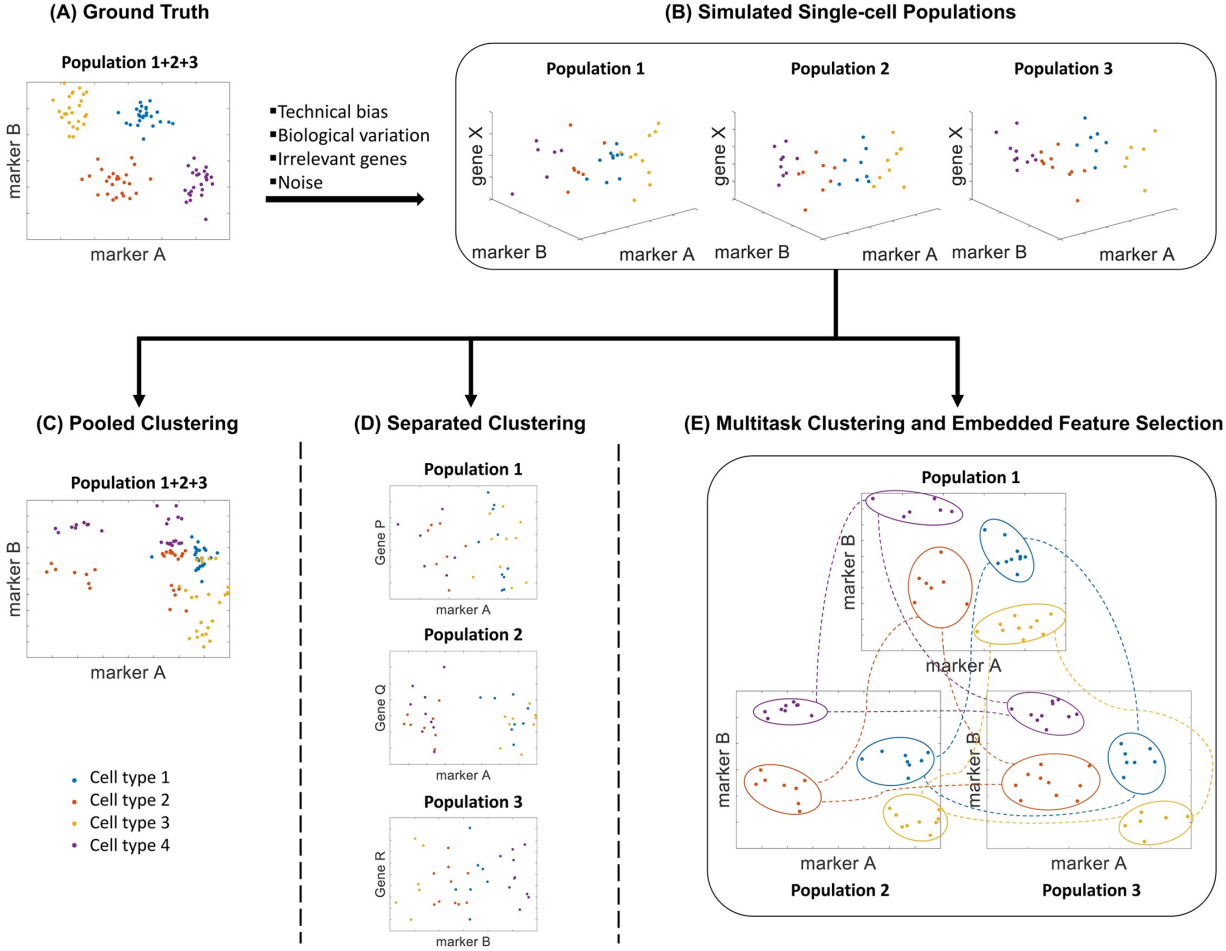
Strategies of clustering multiple single-cell populations. In the example, four cell types are shown in four different colors. **(A)** Ground Truth. 2D plot of a pool of single cells combined from 3 single cell populations of identical distributions separated by the true marker genes A and gene B. **(B)** Simulated Single-cell Populations. 3D plots of the three single-cell populations separated by the marker genes A, B and non-marker gene X. The simulation data are generated from the ground truth data with rotation and scaling to represent technical biases and biological variation with 998 random genes in addition to gene A and gene B (1000 genes in total). Additional noise is also introduced. Three different clustering strategies are shown below in **(C), (D) and (E)**. **(C)** Pooled Clustering. The 2D plot with the true marker genes A and B on pooled data that simply combines 3 single-cell populations together for clustering is shown. Even with the correct marker selection, cells from different types are still mixed because of the rotation, scaling and noise. **(D)** Separated Clustering. The 2D plot on each individual cell population is shown. With the limited single-cell sample size and skewed cell-type distribution, incorrect marker genes may be selected, shown as genes P, Q and R. **(E)** Multitask Clustering and Embedded Feature Selection. The proposed multitask clustering can identify both the true marker genes and correctly cluster the individual cells into their respective types in each population. The clustering of each dataset is reinforced from the results in the other two datasets shown as the connected clusters across the three experiments.

In this paper, we introduce a multitask learning method with embedded feature selection to simultaneously capture the differentially expressed genes among cell clusters and across all cell populations to achieve better single-cell clustering. The key advantage of multitask clustering is the use of multiple single-cell populations to leverage the sample size limitation in each individual dataset while allowing dataset-specific variations among the same cell types across the datasets. To illustrate the objective, Fig 1 shows a simulation example of scRNA-seq data of 100 single cells from three cell populations (n = 33, 33 and 34) with 1000 expressed genes. Among the 1000 genes, gene A and gene B are the hidden markers that are differentially expressed across the four cell types (indicated by four different colors). In the ideal scenario, there is no technical bias and the marker genes are known as shown in the ground truth in Fig 1(A). Fig 1(B) shows the single-cell datasets after biological variation, technical biases, and noise are introduced. The data distributions are very different across the three cell populations after the rotation, re-scaling and addition of noise. It is also challenging to identify the true marker genes with a limited number of samples in each population. Simply pooling the single-cell data from the three populations together will confuse the clustering, even with the correct marker genes identified (Fig 1(C)). Conversely, separated clustering on each single-cell population suffers more from the biological variation as the number of single cells is not sufficient in each individual analysis to identify the true maker genes (Fig 1(D)). As shown in Fig 1(E), variance-driven multitask clustering of single-cell RNA-seq data (scVDMC) utilizes expression patterns of different single-cell populations with shared cell-type markers and corresponding similar clusters for better integration.

## Materials and methods

In this section, we first introduce the model and the algorithm of variance-driven multitask clustering of single cells (scVDMC) and then discuss the parameter selection for scVDMC and related work in scRNA-seq clustering. We also describe the methods for the generation of the in-house Recessive Dystrophic Epidermolysis Bullosa (RDEB) scRNA-seq dataset and the flow cytometry experiments.

### A multitask clustering and feature selection model

**Fig 2:**
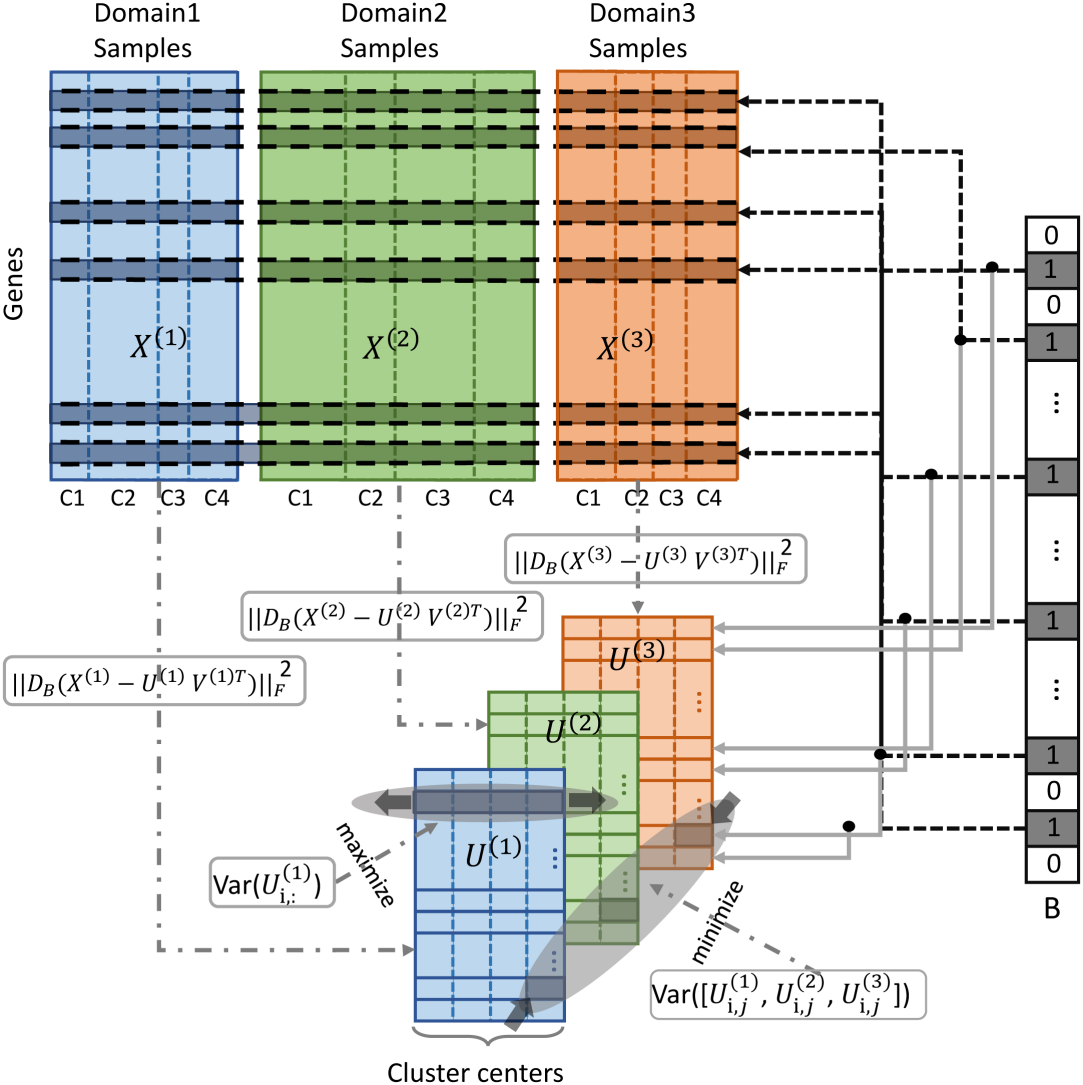
Variance-driven multitask clustering model. Three domains (single-cell populations) are clustered into four cell clusters (C1-C4) in multitask clustering. The samples in each domain are in four clusters separated by the vertical bars. Each dataset is clustered by factorization of the data matrix by the selected genes (with indicator 1 in *B*) common to the three domains. Two types of variance are controlled, 1) the variance among the cluster centers in the same domain are maximized for better cluster separation shown as a shadowed row; and 2) the variance among the shadowed cluster centers across the domains are minimized to match the similar clusters across the domains.

Assume a total of *D* domains with each domain representing a single-cell population for clustering. Let matrix $X^{\left(d\right)}\in R^{m\times n^{\left(d\right)}}$ denote RNA-seq gene expression values from domain *d* ∈ {1, 2, …, *D*}, where *m* is the number of features (genes) and *n*^(*d*)^ is the single-cell sample size of domain *d*. Let $U^{\left(d\right)}\in R^{m\times k}$ denote the cell-type cluster centers, vector $Y^{i,j}={\left[U_{i,j}^{\left(1\right)},U_{i,j}^{\left(2\right)},\ldots ,U_{i,j}^{\left(D\right)}\right]}^{T}$ stack the (*i*, *j*)-th entry of every *U*^(*d*)^ and the binary matrix *V*^(*d*)^ ∈ {0, 1}^*n*^(*d*)^×*k*^ denote the assignments of each single-cell to the clusters, where *k* is the number of cell types (clusters). With the binary vector *B* ∈ {0, 1}^*m*^ denoting the indicators of feature selection (1: selected and 0: not selected) and *D*_*B*_ denoting the diagonal matrix with *B* on the diagonal, scVDMC model outlined in Fig 2 is defined as:

$$\begin{array}{c} \begin{array}{cccc} & \underset{U^{\left(d\right)},V^{\left(d\right)},B}{\text{minimize}} & & \frac{1}{2}\sum_{d=1}^{D}\left|\right|D_{B}\left(X^{\left(d\right)}-U^{\left(d\right)}{V^{\left(d\right)}}^{T}\right){\left|\right|}_{F}^{2}-w\sum_{d=1}^{D}B^{T}\text{Var}\left(U^{\left(d\right)}\right)+\alpha \sum_{i,j}B_{i}\text{Var}\left(Y^{\left(i,j\right)}\right)\\ & \text{subject}\;\text{to} & & \sum B=\lambda ,\\ & & & \sum_{j}V_{i,j}^{\left(d\right)}=1,\forall i=1,2,\ldots ,n^{\left(d\right)},\;\forall d=1,2,\ldots ,D, \end{array} \end{array}$$ (1)

where *w* and *α* > 0 are hyper-parameters to balance the three error terms: the reconstruction error, the cluster center separation in each cell population, and the variance of the cluster centers across the different single-cell populations. $\lambda \in Z^{+}$ is the predefined number of features to be selected. $\left|\right|D_{B}\left(X^{\left(d\right)}-U^{\left(d\right)}{V^{\left(d\right)}}^{T}\right){\left|\right|}_{F}^{2}$ in Eq (1) denotes the reconstruction error of the classic *k*-means clustering as matrix factorization with *D*_*B*_ selecting marker genes by *B*, i.e. the reconstruction error is only measured on the marker genes by ignoring the irrelevant (non-selected) genes. The second term *B*^*T*^Var(*U*^(*d*)^) is introduced to maximize the separation of the cluster centers, where Var(*U*^(*d*)^) is defined as a vector in which each element is the variance of the vector $U_{i,:}^{\left(d\right)}\in R^{k\times 1}$ [5]. The third term Var(*Y*^(*i*,*j*)^) denotes the variance of the vector *Y*^(*i*,*j*)^, which is introduced to require similar gene expression centers across different single-cell populations. Note that the reconstruction error encourages selection of low expression genes since the errors are usually smaller on smaller values while the second variance term encourages selection of high expression genes since the variances tend to be larger on larger values. Together as the sum over all the domains, the cost function provides a balanced error on the compactness and separation of the clusters of the cell types tuned by feature selection across all the domains. The unique but similar cluster centers in each domain preserves the unique expression patterns while the features are selected as common marker genes for different cell types. For the three hyper-parameters in Eq (1), λ (the number of marker genes) is typically a small number based on prior knowledge of the cell types, and the selection of balancing weight *w* and *α* is discussed later in this section.

### Alternating updating algorithm

**Algorithm 1** scVDMC algorithm

1: Input: *X*^(*d*)^, *α*, *k*, *w*, λ, *d* = 1, 2, …, *D*

2: output: *U*^(*d*)^, *V*^(*d*)^, *B*

3: Initialize *U*^(*d*)^ and *V*^(*d*)^.

4: **repeat**

5:  compute *B* with integer linear programming in Eq (7)

6:  **for** *d* = 1, 2, …, *D* **do**

7:   solve *V*^(*d*)^ by Eq (2)

8:   solve *U*^(*d*)^ by (6)

9:  **end for**

10: **until** *U*^(*d*)^, *V*^(*d*)^ and *B* converge

11: **return** *U*^(*d*)^, *V*^(*d*)^ and *B*

The full scVDMC algorithm is shown in Algorithm 1. The goal is to minimize the cost function in Eq (1) to obtain the optimal *U*^(*d*)^, *V*^(*d*)^ and *B*. We employ an alternating update strategy to solve the optimization problem. First, we fix the feature selection *B*, all the cluster centers *U*^(*i*)^, *i* = 1, 2, …, *D* and all other *V*^(*i*)^, *i* ≠ *d*, to obtain a certain *V*^(*d*)^.

$$\begin{array}{c} \begin{array}{cccc} & \underset{V^{\left(d\right)}}{\text{minimize}} & & \frac{1}{2}\left|\right|D_{B}\left(X^{\left(d\right)}-U^{\left(d\right)}{V^{\left(d\right)}}^{T}\right){\left|\right|}_{F}^{2}\\ & \text{subject}\;\text{to} & & \sum_{j}V_{i,j}^{\left(d\right)}=1,\;\forall i=1,2,\ldots ,n^{\left(d\right)}. \end{array} \end{array}$$ (2)

This is equivalent to assigning samples to the nearest centers *U*^(*d*)^ by the Euclidean distance in the features selected by *B*, where each column of *D*_*B*_ *X*^(*d*)^ is a sample and each column of *D*_*B*_ *U*^(*d*)^ is a center. Then the distance of a sample to every center is calculated and the nearest center is chosen to assign 1 to the corresponding *V*^(*d*)^. The time complexity for assigning each sample to one of the *k* clusters over the λ marker genes will be *O*(*n* × *k* × λ), where n is the total number of samples in all the domains.

Next, we fix the feature selection *B*, all clustering assignments *V*^(*i*)^, *i* = 1, 2, …, *D*, and all other *U*^(*i*)^, *i* ≠ *d*, to solve a certain *U*^(*d*)^, rewritten as:

$$\begin{array}{c} \underset{U^{\left(d\right)}}{\text{minimize}}\;\;\frac{1}{2}\sum_{i=1}^{m}B_{i}\left|\right|\left(X_{i,:}^{\left(d\right)}-U_{i,:}^{\left(d\right)}{V^{\left(d\right)}}^{T}\right){\left|\right|}_{2}^{2}-w\sum_{i=1}^{m}B_{i}\text{Var}\left(U_{i,:}^{\left(d\right)}\right)+\alpha \sum_{i,j}B_{i}\text{Var}\left(Y^{\left(i,j\right)}\right), \end{array}$$ (3)

where $\text{Var}(U_{i,:}^{\left(d\right)})$ is the variance of vector $U_{i,:}^{\left(d\right)}$ defined as

$$\begin{array}{c} \begin{array}{cc} \text{Var}(U_{i,:}^{\left(d\right)}) & =\frac{1}{k}\left(U_{i,:}^{\left(d\right)}-\frac{U_{i,:}^{\left(d\right)}1_{k}1_{k}^{T}}{k}\right){\left(U_{i,:}^{\left(d\right)}-\frac{U_{i,:}^{\left(d\right)}1_{k}1_{k}^{T}}{k}\right)}^{T}\\ & =\frac{1}{k}U_{i,:}^{\left(d\right)}\left(I_{k}-\frac{1_{k}1_{k}^{T}}{k}\right){\left(I_{k}-\frac{1_{k}1_{k}^{T}}{k}\right)}^{T}{U_{i,:}^{\left(d\right)}}^{T}\\ & =\frac{1}{k}U_{i,:}^{\left(d\right)}\left(I_{k}-\frac{1_{k}1_{k}^{T}}{k}\right){U_{i,:}^{\left(d\right)}}^{T}, \end{array} \end{array}$$ (4)

where **I**_*k*_ denotes the identity matrix of size *k* and **1**_*k*_ is a length *k* column vector of all ones. Similarly, we have

$$\begin{array}{c} \text{Var}\left(Y^{\left(i,j\right)}\right)=\frac{1}{d}Y^{(i,j)T}\left(I_{d}-\frac{1_{d}1_{d}^{T}}{d}\right)Y^{\left(i,j\right)}. \end{array}$$ (5)

As shown in S1 Appendix, the analytical solution of Eq (3) when *B*_*i*_ = 1 is

$$U_{i,:}^{\left(d\right)}{}^{{}^{T}}={\left(V^{\left(d\right)}{}^{{}^{T}}V^{\left(d\right)}-\frac{2w}{k}\Psi +\frac{2\alpha k\Phi_{d,d}}{d}I_{k}\right)}^{-1}(V^{\left(d\right)}{}^{{}^{T}}X_{i,:}^{\left(d\right)}{}^{{}^{T}}-\frac{2\alpha k}{d}\underset{l\neq d}{\sum }\Phi_{dl}U_{i,:}^{{\left(l\right)}^{T}}).$$ (6)

The time complexity is *O*(*k*^3^) for the matrix inversion and *O*(*n* × *k*^2^) for computing $V^{{\left(d\right)}^{T}}V^{\left(d\right)}$. Since the matrix inversion is common to all the genes and only needs to be computed once, the total time complexity is only *O*(*n* × *k* × λ).

Finally, to update binary vector *B*, we fix all *U*^(*d*)^ and *V*^(*d*)^ to optimize

$$\begin{array}{c} \begin{array}{cccc} & \underset{B}{\text{minimize}} & & \sum_{i=1}^{m}B_{i}(\frac{1}{2}\sum_{d=1}^{D}\left|\right|\left(X_{i,:}^{\left(d\right)}-U_{i,:}^{\left(d\right)}{V^{\left(d\right)}}^{T}\right){\left|\right|}_{2}^{2}-w\sum_{d=1}^{D}\text{Var}\left(U_{i,:}^{\left(d\right)}\right)+\alpha \sum_{j=1}^{k}\text{Var}\left(Y^{\left(i,j\right)}\right))\\ & \text{subject}\;\text{to} & & \sum B=\lambda , \end{array} \end{array}$$ (7)

which is a standard constrained linear binary integer programming problem that can be easily solved by sorting the coefficients of *B* and taking the top λ entries. The time complexity is *O*(*m* × *n* × *k*) for computing the construction error terms, *O*(*D* × *m* × *k*) for computing the variances and *O*(*m* log *m*) for sorting the coefficients. The overall time complexity is *O*(*m* × *n* × *k*) assuming *n* × *k* > log *m*.

Thus, the total time complexity of each iteration in Algorithm 1 will be *O*((*m* + λ) × *n* × *k*), which is comparable to *k*-means when λ <<*m*.

### Parameter selection

There are four hyper-parameters to tune for the scVDMC algorithm, *α* and *w*: weights of the two variance terms, *k*: the number of clusters and λ: the number of marker genes. Below we describe our strategies for tuning *α*, *w* and *k* assuming that λ can be approximately informed by prior knowledge of the cell types.

**Tuning *α*:** The role of *α* is to weight the cost term on the cross-domain variance of the cluster centers. The larger the *α* the more similar the cluster centers are across the domains. Ideally, *α* should be relatively small to allow smaller reconstruction error but yet meet the consistency requirement across the domains. The strategy is to start with a small *α* and measure the total difference between the cluster centers of the corresponding cluster across the domains, and then increase *α* to reduce the difference until the total difference does not change much. This selection can also be achieved by visualization of the cluster centers with Principle Component Analysis (PCA) or other dimension reduction methods. After clustering, we can project the data in each domain into the first two PCs. The distance between the cluster centers of the same cluster in each domain can be compared for choosing an appropriate *α*. Several examples are shown later in the experiments.

**Deriving the upper bound of *w*:** Eq (3) is a sum of a few quadratic terms of variable $U_{i,:}^{\left(d\right)}$. The global minimum of $U_{i,:}^{\left(d\right)}$ can be solved in closed-form if the Hessian below is positive semi-definite,

$$H={V^{\left(d\right)}}^{T}V^{\left(d\right)}-\frac{2w}{k}\Psi +\frac{2\alpha k\Phi_{d,d}}{d}I_{k}.$$ (8)

In the following, we show that an upper bound on *w* will guarantee that *H* is positive semi-definite. By Gershgorin circle theorem (For any eigenvalue *δ* of matrix *H*, |*δ* − *H*_*ii*_| ≤ ∑_*j*≠*i*_ |*H*_*ij*_| for ∀*i* ⇔ *H*_*ii*_ − ∑_*j* ≠ *i*_ |*H*_*ij*_| ≤ *δ* ≤ *H*_*ii*_ + ∑_*j* ≠ *i*_ |*H*_*ij*_|.), the sufficient condition of *H* ≽ 0 is *H*_*ii*_ − ∑_*j* ≠ *i*_ |*H*_*ij*_| ≥ 0 for ∀*i*. This is equivalent to stating that *H* is diagonally dominant and only has non-negative diagonal entries. *H* can be rewritten as follows,

$$\begin{array}{cc} H_{ii} & =c_{i}+\frac{2w(1-k)}{k^{2}}+\frac{2\alpha k(d-1)}{d^{2}},\;\forall i=1,...,k\\ H_{ij} & =\frac{2w}{k^{2}},\;\forall i\neq j, \end{array}$$

where *c*_*i*_ is the *i*^*th*^ diagonal entry of matrix $V^{{\left(d\right)}^{T}}V^{\left(d\right)}$, i.e., the size of cluster *i*. Then we have

$$c_{i}+\frac{2w(1-k)}{k^{2}}+\frac{2\alpha k(d-1)}{d^{2}}\geq \frac{2w(k-1)}{k^{2}}$$

and thus,

$$\begin{array}{c} w\leq \frac{k^{2}c_{min}}{4(k-1)}+\frac{\alpha k^{3}\left(d-1\right)}{2d^{2}\left(k-1\right)} \end{array}$$

where *c*_*min*_ is the minimum of *c*_*i*_, ∀*i* = 1, …, *k*. Since *c*_*min*_ ≥ 1 (no empty cluster), we obtain a loose upper bound of $w=\frac{k^{2}}{4(k-1)}+\frac{\alpha k^{3}\left(d-1\right)}{2d^{2}\left(k-1\right)}$. In all the experiments, we set *w* to be smaller than the upper bound for feasible implementation.

**Determining the number of clusters *k*:** The number of clusters *k* is selected by an “elbow” plot of the within-clusters sum of squares *T*_*s*_ computed as follows:

$$\begin{array}{c} T_{s}=\sum_{d=1}^{D}\left|\right|D_{B}\left(X_{i,:}^{\left(d\right)}-U_{i,:}^{\left(d\right)}{V^{\left(d\right)}}^{T}\right){\left|\right|}_{2}^{2}. \end{array}$$ (9)

*T*_*s*_ represents the amount of variance to minimize for better clustering. Larger *k* will lead to smaller *T*_*s*_. By plotting *T*_*s*_ under different options of *k*, we can select the best *k* at the so-called “elbow” of the curve. S6 and S7 Figs show the “elbow” plot on two datasets in the experiments. In addition, when an empty cluster is created, the calculation of cluster center variance will be invalid. To address the possible issue, we use a simple splitting procedure to handle empty clusters. Specifically, if there is an empty cluster in *V*^(*d*)^ (i.e. the whole column is 0) we randomly split the largest cluster into two clusters. This procedure is repeated until there are exactly *k* clusters. This strategy is similar to commonly used *k*-mean rerun when a cluster center is collapsed on a single data point or no data point.

### scRNA-seq of RDEB cohort

To identify sub-populations producing homing signals that could attract bone marrow-derived cells to injured skin, we captured single dermal fibroblasts from six patients with severe generalized RDEB and their HLA-matched healthy siblings using the Fluidigm C1 system. The demographics information of the patients and donors are shown in S1 Table.

**Cell culture:** Dermal fibroblasts from patients with severe generalized RDEB and their human leukocyte antigen (HLA) matched healthy siblings were obtained from skin biopsies and cultured in DMEM high glucose (Thermo Fisher Scientific) containing 10% fetal bovine serum (MilliporeSigma), 1% Pen/Strep (Thermo Fisher Scientific), 1% L-glutamine (Thermo Fisher Scientific), and 1% MEM NEAA (Thermo Fisher Scientific). For sub-culture, the medium was removed and cells were washed with 1X PBS (Thermo Fisher Scientific) and detached using Trypsin/EDTA (Thermo Fisher Scientific). Experiments were performed with fibroblasts at passages 4-9.

**Single-cell capture and RNA-seq:** Fibroblasts were collected by trypsinization and resuspended in 5 *μ*L of fibroblast medium for loading into the capture chip. The medium- (10-17 *μ*m diameter) and large-size (17-25 *μ*m diameter) chips were used to capture cells with the C1 system (Fluidigm). Cells were loaded at a concentration of 2.5 x 10^5^ per *μ*L and stained with the Live/Dead Viability/Cytotoxicity kit (Thermo Fisher Scientific). Cells were imaged with phase-contrast and fluorescence microscopy to assess cell number and viability at each capture point. Capture sites with single, live cells were selected while capture sites with multiple, no, or an unclear number of cells were excluded from further analysis. Images for each single-cell used in this study are available upon request. In total, 295 patient cells and 248 sibling cells were selected. On the device, cDNA was created from the selected cells using the SMARTer Ultra Low RNA kit designed for the C1 system (Clontech). mRNA libraries were constructed using the Nextera XT kit (Illumina) according to the manufacturer’s protocol. The libraries were sequenced on an Illumina MiSeqv3 with 75bp paired-end reads to a depth of 19-22 million reads per lane. Target sequencing depth for each library was 200K reads. The RNA-seq data have been deposited in NCBI’s Gene Expression Omnibus and are accessible through GEO series accession number GSE108849 (https://www.ncbi.nlm.nih.gov/geo/query/acc.cgi?acc=GSE108849).

**Processing of RNA-seq data:** Paired-end 75bp reads were mapped to the UCSC human transcriptome (hg19) using Bowtie2 (version 2.2.4) and Tophat (version 2.0.9). Gene expression levels were calculated using Cuffquant (Cufflinks version 2.2.1 with parameters -u -max-bundle-frags 10000000) and Cuffnorm (Cufflinks version 2.2.1). FPKM values as estimated by Cufflinks were added a value of 1 (to avoid zeros) and log2 transformed. We removed nine single-cell samples with low read counts (< 50K) and sub-sampled two single-cell samples sequenced as population controls with high read counts (> 1.5M) (random sub-sampling, 10% of total reads). 11 single-cell samples were excluded as outliers. We excluded lowly expressed genes (average log2 (FPKM) < 1.5) from further analysis. The remaining 543 single-cell samples met the requirement of expressing at least 2,000 of these remaining 5,196 genes. For each individual, the number of single-cells used in the analysis and the average number of reads for those single-cells is summarized in Table 1. The total number of the reads and the number of aligned reads in each cell are also shown in S3 Fig.

**Table 1:** For each RDEB or WT individual, the number of single-cells used for downstream analysis is indicated as well as the average number of reads for the single-cells from each individual.

RDEB-WT pairs	RDEB cells	Avg. reads	WT cells	Avg. reads
1	41	248,929	20	205,216
2	72	200,961	46	241,966
3	54	138,598	37	146,610
4	36	83,513	51	86,483
5	46	181,263	47	176,929
6	46	175,346	47	170,307

**Flow cytometry:** Fibroblasts were collected by trypsinization (as above) and resuspended in fibroblast medium. A BD Cytofix/Cytoperm^™^ kit (BD Biosciences) was used to prepare the cells for intracellular staining. Cells were fixed for 15 min with 150 *μ*l Fixation/Permeabilization solution before being resuspsended in 300 *μ*l 1X BD Perm/Wash Buffer and incubated at 4°C for 20 min. Primary antibodies (S2 Table) were diluted in 100 *μ*l 1X BD Perm/Wash Buffer and cells were resuspended in this for 20-30 min at 4°C, followed by one wash with 500 *μ*l 1X BD Perm/Wash Buffer. Secondary antibodies (S3 Table) were diluted in 300 *μ*l 1X BD Perm/Wash Buffer and cells were resuspended in this for 20-30 min at 4°C, followed by one wash with 500 *μ*l 1X BD Perm/Wash Buffer, and resuspension in 300 *μ*l 1X BD Perm/Wash Buffer. Flow cytometry experiments were carried out on a BD LSRII system equipped with FACsDiva 8.0 software (BD Biosciences) and analyzed using FlowJo (Tree Star Inc.).

### Related work

Most existing methods focus only on sub-population clustering and differential gene expression detection among the learned cell clusters with one (pooled) cell population. Some of these methods were directly adopted from traditional bulk RNA-seq analysis and/or classical dimension reduction algorithms such as Principal Component Analysis [6–8], hierarchical clustering [9], t-SNE [10–12], Independent Component Analysis [13] and Multi-dimensional Scaling [14]. Other methods focus on special properties of scRNA-seq data, such as high variance and uneven expressions. For example, SNN-Cliq [15] uses a ranking measurement to get reliable results on high dimensional data; [16] proposed a special dimension reduction method to handle the large amount of zeros in scRNA-seq; [17] proposed a Latent Dirichlet Allocation model with latent gene groups to measure cell-to-cell distance; CellTree method [17] clusters single cells by a detected tree structure outlining the hierarchical relationship between single-cell samples to introduce biological prior knowledge; Seurat [18] was proposed to infer cellular localization by integrating single-cell RNA-seq data with *in situ* RNA patterns; and more recently a consensus clustering approach SC3 [19] was proposed to improve the robustness of clustering through combining multiple clustering solutions by consensus.

Mixed multiple batch strategies [9, 20] have been proposed to reduce the technical variance, which does not directly improve clustering. To the best of our knowledge, multitask clustering with an embedded feature selection has not been previously applied to scRNA-seq data analysis.

### Ethics approval and consent to participate

All patients gave consent for samples to be taken per the Declaration of Helsinki. This research was approved by the University of Minnesota’s Institutional Review Board: IRB 1301M26601: MT2013-02R (Establishment of a Cell and Tissue Repository for Human Cell Reprogramming and Derivation of iPS Cell Lines to Investigate Mechanisms and Treatment of Human Disease).

## Results

In the experiments, scVDMC was applied to two small scRNA-seq datasets: mouse embryonic stem cell (mESC) data [21] and mouse embryonic lung epithelial cell (Lung) data [22], and one large-scale droplet-based scRNA-seq peripheral blood mononuclear cells (PBMC) data [23]. We also applied scVDMC to our in-house Recessive Dystrophic Epidermolysis Bullosa (RDEB) data to detect RDEB relevant cell types and marker genes. The statistics of the four datasets are shown in Table 2.

**Table 2:** Four datasets used in the experiments.

Datasets	# of cells	# of clusters	# of domains	# of cells in each domain
mESC	250	3	3	81:90:79
Lung	77	4	3	20:34:23
PBMC	27,302	10	3	10000:7783:9519
RDEB	543	4	6	61:118:91:87:93:93

### Experimental design

scVDMC was compared with six baseline methods: (1) *k*-means clustering on each domain separately, (2) pooling all domains and applying *k*-means clustering, (3) SNN-Cliq [15], (4) CellTree [17], (5) Seurat [18] and (6) SC3 [19]. Pooled *k*-means (2) was used to obtain the initialization for scVDMC.

To apply the SNN-Cliq method [15], we used the provided MATLAB code to transform the data into the SNN graph, then used the Python code to produce the clustering result by ranking measurement. There are three hyper-parameters: *k* (size of the nearest neighbor list), *r* (parameter for quasi-clique finding, range (0,1]), and *m* (parameter for cluster merging range (0,1]). We tested multiple combinations of the three hyper-parameters using *k* = 3, 5, 7, *r* = 0.1, 0.2, …, 0.9 and *m* = 0.1, 0.2, …, 0.9. We also required the program to annotate all the data instead of leaving singletons unlabeled (−*n*). Since SNN-Cliq identifies the number of clusters automatically, we only reported the results with the correct number of clusters. In all experiments with SNN-Cliq, we further removed genes with low expression and log-transformed the data, as recommended in [15].

To apply the CellTree method [17], we used the provided R package to first fit a Latent Dirichlet Allocation (LDA) model with the default method (joint MAP estimation) to choose the number of topics followed by learning a pair-wise distance for all cells. Then we ran hierarchical clustering with four different methods for computing cluster distance (‘ward’, ‘complete’, ‘single’, ‘average’) and selected the best clustering results.

To apply Seurat [18], Seurat v2.0 R package was downloaded from SATIJA LAB. The scRNA-seq data were converted into the required format (gene index | cell index | gene expression) as the input. The parameter “Resolution” tunes the granularity of the downstream clustering, with increased values resulting a larger number of clusters. We tested a range [0.5,1.5] to get the exact number of clusters for comparison with other methods. The reported result of Seurat is computed with the resolution parameter that gives the exact number of clusters and the lowest error.

To apply the SC3 [19] we downloaded SC3 v1.7.2 R package from Bioconductor. All parameters in SC3 are set to default. In the experiments with more than 5000 instances for clustering, the SVM mode will be trigged to run a second stage supervised learning to improve the scalability.

To further test separated cluster, pooled clustering and SC3 combined with feature selection, we chose the genes with larger variance as the marker genes. Since the other three baselines use a different strategy for clustering and do not provide marker-gene selection, we only focused on the clustering result for these three baselines. The true cluster labels are obtained as the validated clusters with high confidence in the mESC data [21] and Lung data [22], and the known PBMC populations from donor A sorted with FACS analysis [23].

### Experiment on mouse embryonic stem cell data

We downloaded the single-cell expression data for 250 mESCs [21] from the European Bioinformatics Institute’s (EBI) ESpresso database. These 250 mESCs cultured in serum conditions were captured using the Fluidigm C1 on three different days from three different passages (biological replicates, n = 81, 90, and 79). After removing genes expressed uniformly within a single replicate, 12,114 genes remained. To tune *α* for scVDMC, we examined the positions of the cluster centers across the domains and show the visualization by PCA in S4(A) & S4(B) Fig. Based on the visualization, *α* = 0 and 1 are chosen since the relative positioning of cluster centers are similar in all the three domains. For the SNN-Cliq method, we further removed genes with log-transformed average expression less than 20.

**Fig 3:**
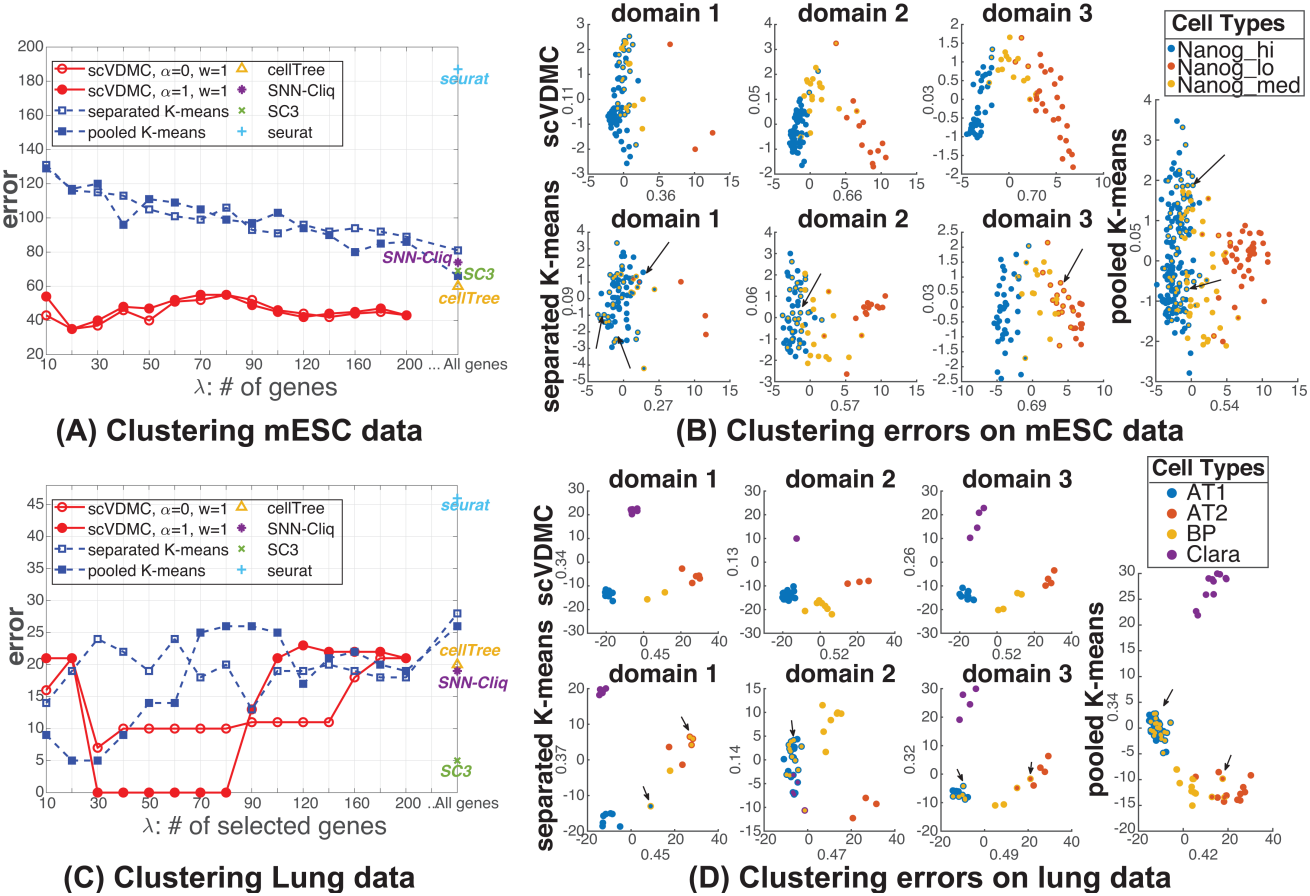
Clustering performance on mESC and Lung datasets. (A) & (C) show the clustering results of the scVDMC algorithm compared with the baseline methods. Pooled *k*-means, separated *k*-means and scVDMC are tested with varying numbers of selected marker genes. Seurat, cellTree, SNN-Cliq and SC3 are tested using all the genes as input to the software/program. (B) & (D) show the PCA of scVDMC, pooled *k*-means, and separated *k*-means results on the selected top marker genes. PCA is applied on each individual domain for separated *k*-means and the combined data for pooled *k*-means and scVDMC. For each dot, the layer (outer) color indicates the true cell type, while the inner color indicates the predicted cell type. The error is measured on the best one-to-one matching between the detected clusters and the true clusters. The hyper-parameters for scVDMC are λ = 20, *w* = 0.1, *α* = 0.5 on the mESC dataset and λ = 50, *w* = 0.1, *α* = 1 on the Lung dataset.

Fig 3(A) shows the clustering results. Compared with the six baselines, scVDMC shows a consistently lower error with different choices of λs. Within a reasonable range of λ, such as from 20 to 200, scVDMC shows significant improvement compared with the baseline methods. When λ is too small, such as 10 genes selected, there are not enough markers to capture the difference among the cell types such that the error is larger. When λ is too big, scVDMC will consider almost all the genes and the variance selection will not play a role. As such, scVDMC will eventually degrade into separated *k*-means and the error will also increase. As shown in S1(A) Fig, it is worth noting that the results are less sensitive to the choice of the parameter *w*, for which the upper bound for *w* is $\frac{9}{8}$ in this case. It is also interesting that the CellTree method performed better than both pooled and separated *k*-means, while SNN-Cliq and SC3 performed better than separated *k*-means but worse than pooled *k*-means. Under various tuning of the parameters, Seurat still performed poorly on this dataset. Both separated *k*-means and pooled *k*-means performed much worse with the feature selection by variance, indicating that simple feature selection strategies will not identify correct markers in this dataset. Running scVDMC with *α* = 1 performed the best when 20 marker genes are selected but the overall performance is very similarly as running with *α* = 0, indicating that the control of the cross-domain variance could play a role in improving the results. However, since the cluster centers are already not very different when running with *α* = 0, the improvement will only be marginal. Fig 3(B) shows the detailed clustering errors by scVDMC, pooled *k*-means and separated *k*-means. Compared with the pooled *k*-means and separated *k*-means, scVDMC captures relatively high variance in the leading principle components and achieves improved clustering in every domain (fewer mixed-color dots). In S2(A) Fig, we also show the convergence of scVDMC by the number of iterations.

Analysis of the mESC transcriptome data using scVDMC yielded comparable results on marker gene selection in the original paper [21] as well as pooled and separated *k*-means. Both analyses were able to detect and highly rank the known markers of differentiation *Krt8, Krt18, Anxa1, Anxa3*, and *Acta1*. Further, scVDMC detected several additional genes that pooled *k*-means, separated *k*-means and the original paper did not. These included *Cav1*, which is required for normal lung development [24] and *Dsp*, variants of which are associated with idiopathic pulmonary fibrosis [25].

### Experiment on lung epithelial single-cell data

We downloaded the single-cell expression data for 80 embryonic mouse lung epithelial cells [22]. These 80 single-cell samples were taken from three different mice (biological replicates, n = 20, 34, and 23) and contained five cell types: ciliated, Clara, AT1, and AT2 cells, as well as a bi-potential progenitor (BP). Since only one replicate contained ciliated cells, we removed these from the analysis, leaving 77 single-cell samples. After removing genes expressed uniformly within a single replicate, 7,357 genes remained. To tune *α* for scVDMC, we examined the positions of the cluster centers across the domains and show the visualization by PCA in S4(C) & S4(D) Fig. *α* = 1 is chosen as the optimal parameter to achieve similar relative positioning of cluster centers in all the three domains. For the SNN-Cliq method, we further removed genes with log-transformed average expression less than 2.

With the limited number of single-cell samples in this dataset, scVDMC still much improved clustering over the baselines in the range of λ ∈ [30, 80] shown in Fig 3(C). In Fig 3(D), PCA plots of the top 50 genes show a trend similar to the ESC dataset, where scVDMC’s top genes capture more variance and show less clustering error. Both SNN-Cliq and CellTree performed better than pooled *k*-means and separated *k*-means, with SNN-Cliq leading CellTree by a very small margin. Similarly, Seurat also performed poorly while SC3 performed well on the dataset with only 5 mistakes. It is also interesting to observe that running scVDMC with *α* = 1 performed significantly better than running with *α* = 0, indicating that the control of the cross-domain variance played an important role in improving the results. Since the cluster centers are very different when running with *α* = 0, the improvement is significant. Another interesting observation is that the clustering performance is more sensitive to the number of marker genes to select by scVDMC. In particular, selection of 20-80 genes with scVDMC (*α* = 1) will give the optimal clustering results while selection of more than 90 genes will give much higher error. This is due to the small clusters in this dataset (e.g. purple cluster in domain 2 and yellow cluster in domain 1), which could be sensitive to the number of selected genes in low-read-coverage samples. Thus, the error will be more sensitive to the gene selection in this small dataset. On this dataset, both separated *k*-means and pooled *k*-means performed better with the feature selection by variance but never achieved zero clustering error as scVDMC does. As shown in S1(B) and S2(B) Figs, scVDMC behaved similarly by the choices of the *w* parameters and the convergence.

Analysis of the mouse lung epithelial transcriptome data using scVDMC yielded comparable results in the original paper [22] as well as pooled and separated *k*-means. Both analyses were able to detect and highly rank the known marker genes of the different cell types: Clara (*Scgb1a1*), AT1 (*Pdpn, Ager*), and AT2 (*Sftpc, Sftpb*). Further, scVDMC detected several additional genes that pooled *k*-means, separated *k*-means and the original paper did not. These included two components of the Notch signaling pathway (*Notch1* and *Nrarp*) previously shown to be critical for the development of lung alveolar spaces, with AT2 cells being major sites of Notch activation [26].

### Experiment on peripheral blood mononuclear cells data

We downloaded the peripheral blood mononuclear cells (PBMC) data generated by [23] from the 10xGenomics website. In the original data, there are 10 bead-enriched subpopulations of PBMC from a fresh donor (Donor A) with 93802 cells in total. In addition, there are also PBMC from two other frozen donors (Donor B and C) with 7783 and 9519 cells, respectively. A massive droplet-based method was applied to count the mRNAs in the tens of thousands of cells in parallel. To better evaluate the multitask learning setting, we sampled from each of the 10 subpopulations of Donor A in proportion to the sizes of the populations to obtain four subsets of cells from Donor A with 200, 500, 1000 and 10000 cells by sampling. We repeated the sampling procedure five times to generate the mean and variance of Adjusted Rand index (ARI) [19]. We kept all the cells in Donor B and C. We removed the genes expressed in less than 3 cells which results in 17647 genes remained.

To determine the number of clusters in the PBMC data, we examined the “elbow” plot in all the three cell populations shown in S6 Fig. The plots show consistent patterns in the three cell populations that the “elbow” is observed to start around *k* = 10 verifying that there are indeed around 10 cell types in the data. To tune *α* for scVDMC, we examined the positions of the cluster centers across the domains and show the visualization by PCA in S4(E) & S4(F) Fig. *α* = 5 is chosen since the relative positioning of cluster centers are also relatively similar in the three domains. The baseline methods *k*-means and SC3 are tested on the pooled data (mixture of Donor A, B and C) and separated data (Donor A only). For SC3, the hybrid approach (consensus clustering + SVM) with its default parameters is applied on the pooled data due to the scalability issue [19]. Clustering performance is measured using Adjusted Rand index (ARI) [19] by comparing the predicted labels with the true labels from sampling the ten subpopulations of PBMC in Donor A.

**Fig 4:**
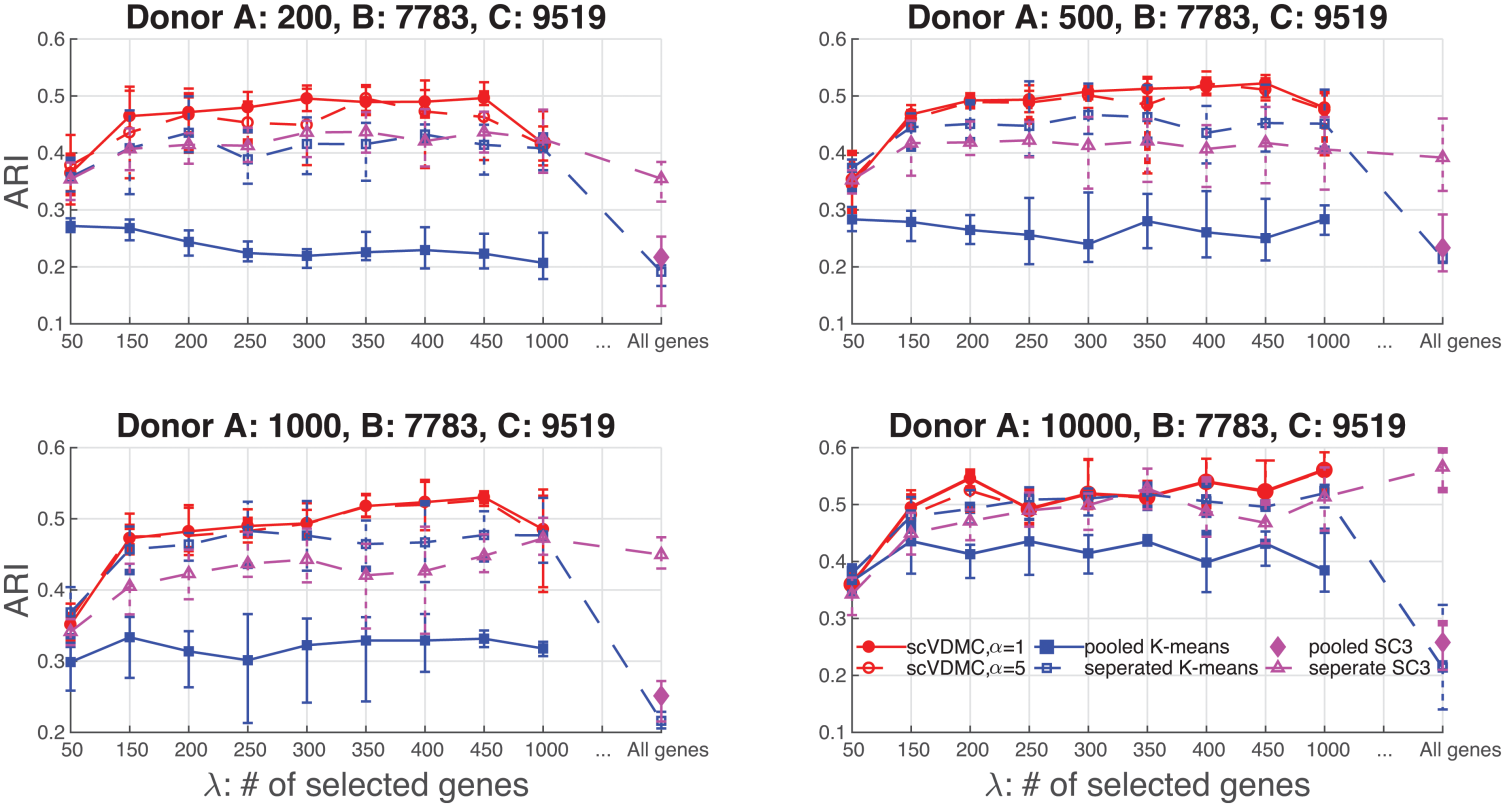
Clustering performance on PBMC dataset. The clustering performance of scVDMC compared with the baseline methods on the single cells from donor A measured by adjusted rand index (ARI). Pooled *k*-means, separated *k*-means, SC3 and scVDMC are tested with varying numbers of selected marker genes. Separated *k*-means Seurat, cellTree, SNN-Cliq and SC3 are tested using all the genes as input to the software/program. To show the strength of multitask learning, different numbers of cells, 200, 500, 1000 and 10000, are sampled from the donor A data and combined with the 7783 cells from donor B and 9519 cells from donor C for clustering. The hyper-parameters for scVDMC are *w* = 0.5, *α* = 1 or 5.

Fig 4 shows the clustering results. Compared with pooled *k*-means and SC3, scVDMC shows a consistently higher ARI with different choices of λs. scVDMC also shows a significant improvement compared with separated *k*-means and SC3 when there are 200, 500 and 1000 cells from Donor A. The improvement by scVDMC becomes only marginal when there are 10000 cells sampled from Donor A. The observation is common since larger dataset often benefit less from multitask learning, i.e. as the sample size in donor A increases, less additional information carried in the data of donor B and C can inform a better clustering of donor A data. On this dataset, we also observed that the clustering performance of scVDMC does not rely on the parameter *α*. This is likely because the agreement among the 10 clusters in the three domains is already high when *α* = 0 as shown in S4(E) Fig. Therefore enforcing stronger agreement by increasing *α* will not lead to big improvement as shown in S4(F) Fig. Overall, scVDMC performed well on the large-scale data showing the advantage of applying multitask learning. SC3 did not over-perform separated *k*-means indicating the consensus clustering is less effective on this dataset.

### Case study of RDEB scRNA-seq data

Recessive Dystrophic Epidermolysis Bullosa (RDEB) is an inherited blistering disorder caused by loss-of-function mutations in the *COL7A1* gene that codes for type VII collagen (C7) [27]. C7 forms the anchoring fibrils that attach the epidermis to the dermis [28]. When C7 is missing, the skin becomes extremely fragile, eroding at the slightest touch. From birth, patients with this disease must undergo intensive bandaging and daily wound care. They are also susceptible to a highly aggressive form of squamous cell carcinoma [29–32]. It has been shown that allogeneic hematopoeitic cell transplant (HCT) can partially rescue the RDEB phenotype. Cells from the bone marrow home to the skin and deposit C7 at the dermal-epidermal junction, greatly improving skin integrity in a subset of patients [33]. However, the molecular mechanism by which this occurs remains unknown.

To determine the number of clusters in the RDEB data, we examined the “elbow” plot in all the six cell populations shown in S7 Fig. The plots show consistent patterns in all six cell populations that the “elbow” starts from *k* = 4, which was chosen as the number of clusters for clustering in all the experiments on the RDEB data. The convergence of scVDMC on RDEB data is shown in S2(D) Fig.

**Fig 5:**
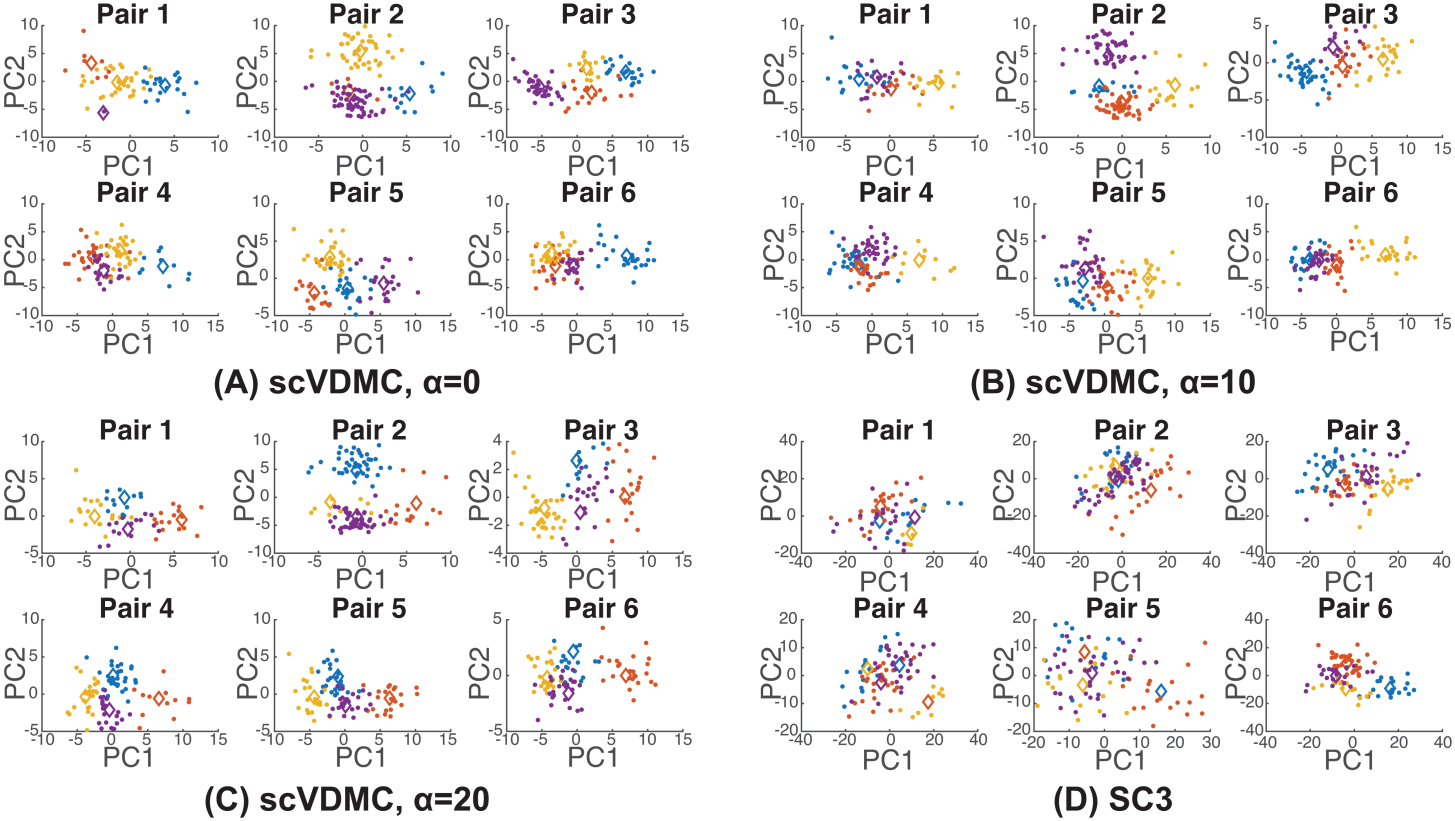
Distinct single-cell populations from six RDEB patients and their matched siblings. In (A), (B) and (C) PCA is applied to the combined single cell profiles of the learned marker genes by scVDMC from the six cell populations. parameters *α* = 0, 10 and 20 are tested. (D) PCA is applied to the combined single cell profiles of all the genes from the six cell populations and the clusters are found by SC3 are shown. Each plot shows the projection by the first two principle components. The cluster centers are indicated by the diamonds.

Applying scVDMC to the RDEB single-cell dataset revealed quite different cell population structures for the six patient-sibling pairs. As shown in Fig 5, the agreement among the cluster centers across the six populations varies under different choices of *α*. When *α* = 0, no agreement among the cluster centers are required. The arrangement of the four cluster centers are very different in the six populations (Fig 5(A)). With larger values of *α*, the arrangement of the cluster centers becomes more similar. When *α* = 20, the structure of the four cluster centers is almost identical for the six populations (Fig 5(C)). The visualization in Fig 5 clearly illustrates the effect of imposing variance constraint on the cluster centers across the populations to account for the population specificity and commonality. For comparison, we also applied SC3 on the pooled cell populations and the individual cell populations. SC3 failed to detect any cluster structures in the pooled cell populations by simply clustering the cells based on the sample origin as shown in S5 Fig. SC3 also only detected inconsistent clusters across the six populations as shown in Fig 5(D) as expected since SC3 unlike scVDMC only clusters the cell populations independently.

**Fig 6:**
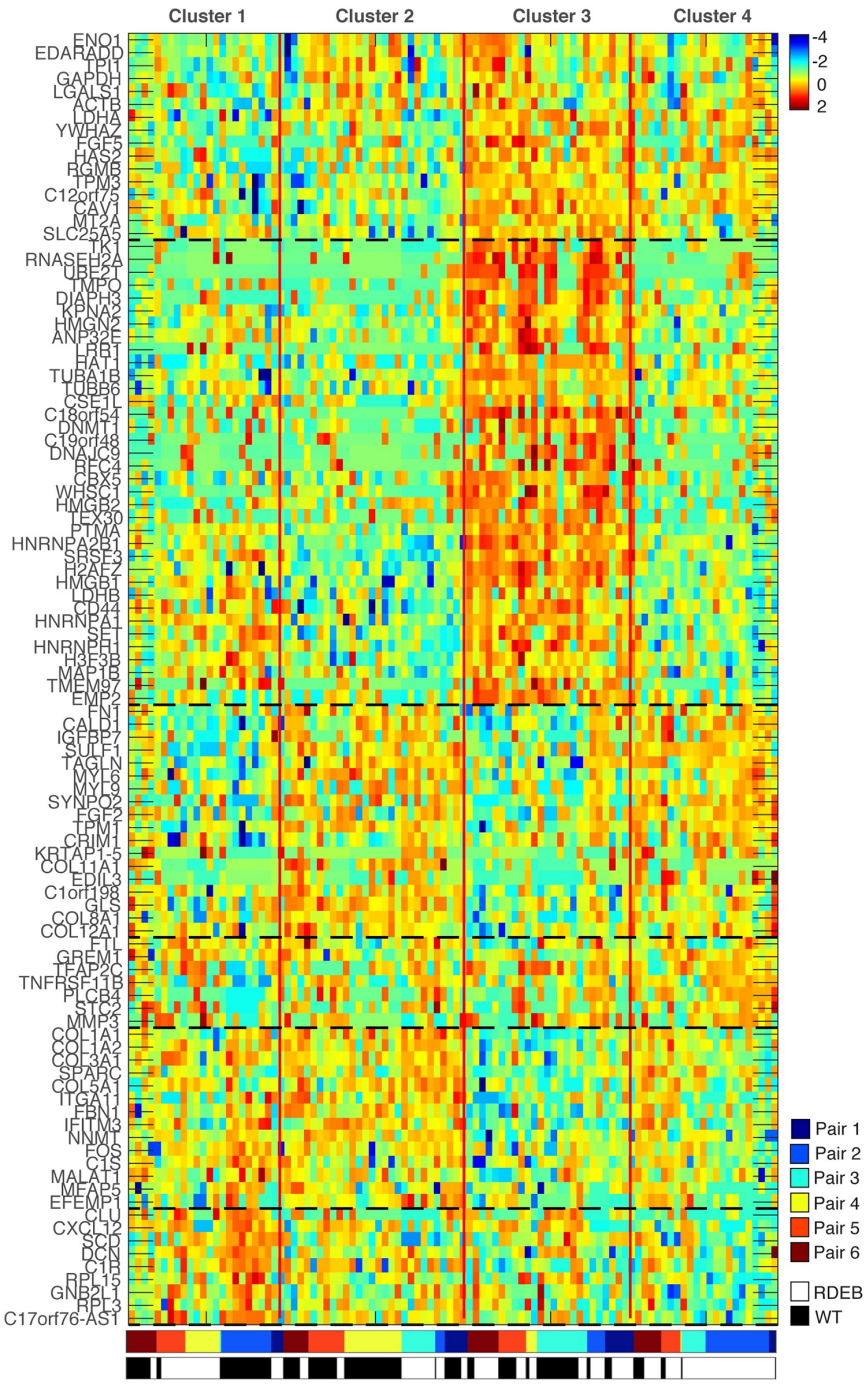
Single-cell clustering by 100 markers genes on the RDEB data with scVDMC. The solid vertical red lines separate the cell clusters and the black dashed horizontal lines indicate marker gene clusters derived by hierarchical clustering. The sample origin of the single cells are also annotated at the bottom by the color bars.

scVDMC identified several marker genes previously known to be involved in RDEB (Fig 6). These included *CXCL12/SDF1*, the ligand for *CXCR4*, which directs cells of the bone marrow to damaged tissue including skin [34] and *HMGB1*, which has shown to be positively correlated with RDEB severity [35] and also mediates recruitment of bone marrow-derived cells to injured tissue [36]. Note that we empirically removed confounding cell cycle genes from the top 100 predicted markers and repeated scVDMC until there were no selected cell cycle genes.

**Fig 7:**
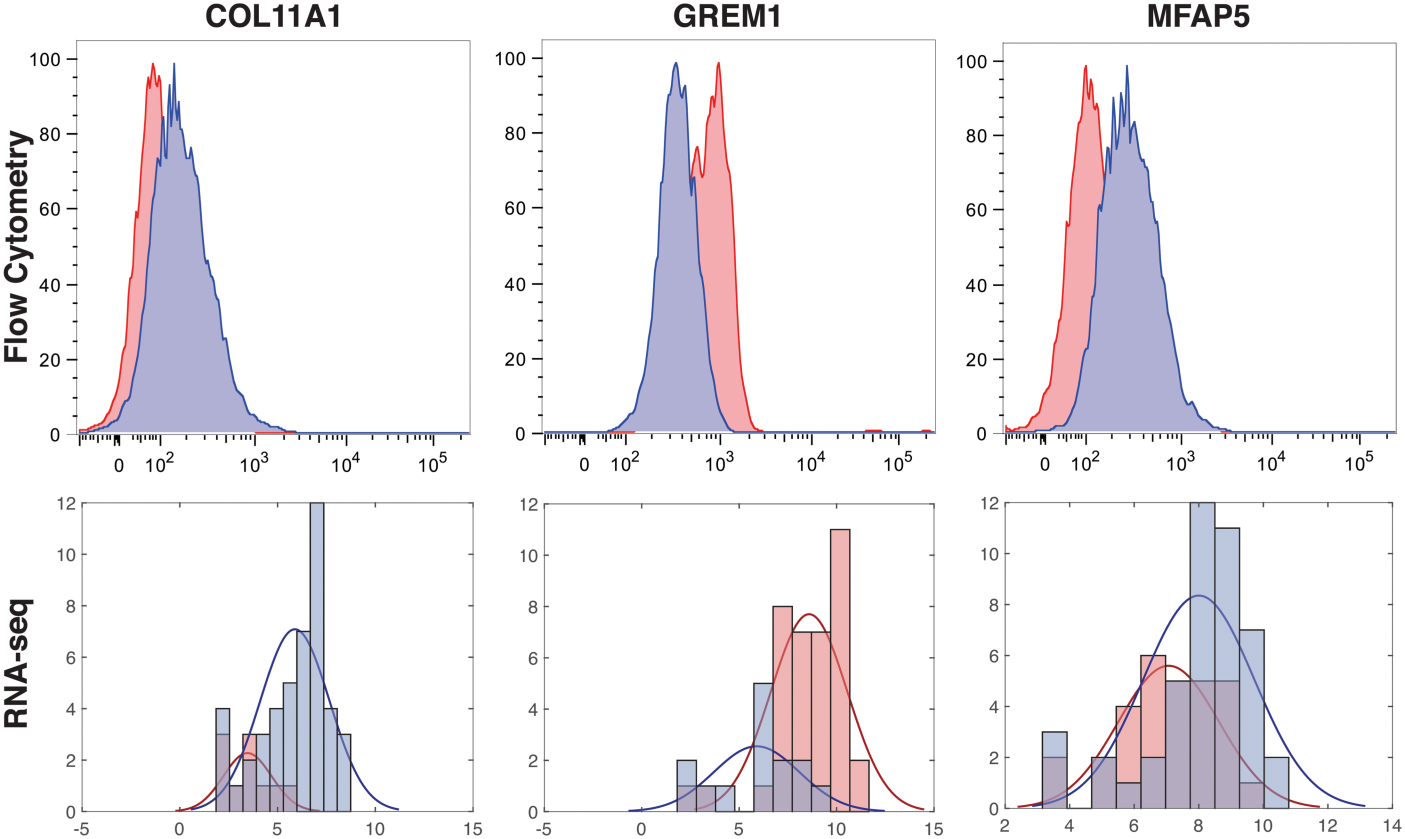
Validation of the novel markers by flow cytometry. The distribution of expressions for novel genes was similar between flow cytometry experiments (top) and the single-cell RNA-seq data (bottom) for the genes *COL11A1, GREM1,* and *MFAP5*. RDEB patient single-cells are shown in red; matched sibling single-cells are shown in blue. Flow cytometry data are measured as percent of max; RNA-seq data measured in FPKMs. RDEB-WT Pair 4 shown for *COL11A1* and *MFAP5*; RDEB-WT Pair 1 shown for *GREM1*.

**Fig 8:**
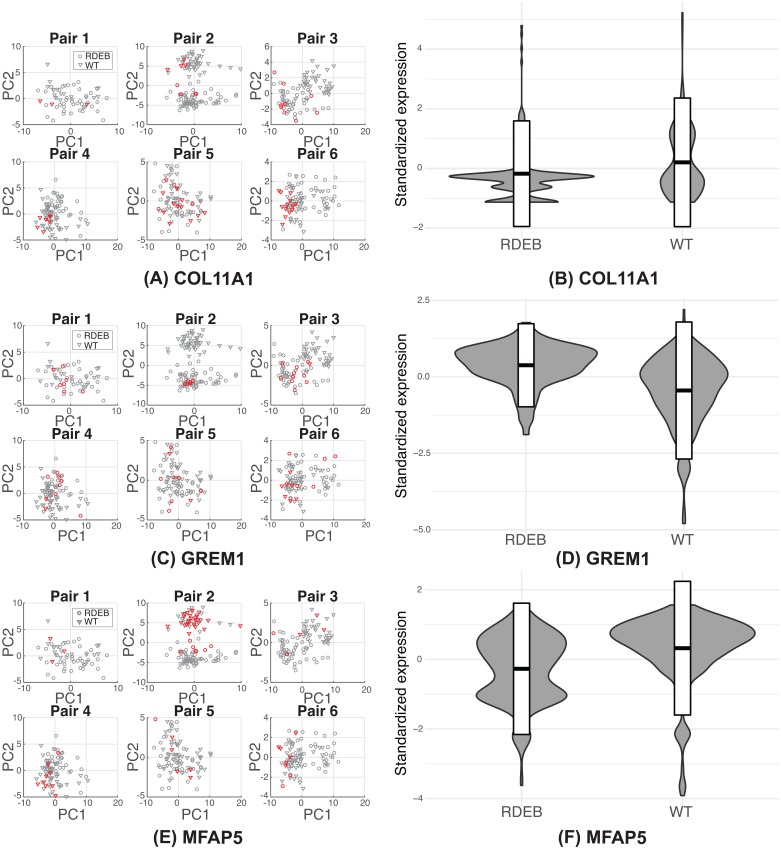
The expressions of the markers genes in the RDEB cells and WT cells. The scatter plots in (A), (C) and (E) show the single cell profiles of the top-100 genes projected to the first two principal components obtained by PCA with the circles representing RDEB cells and triangles representing WT cells. The cells with highly expressed markers are marked in red. The violin plots in (B), (D) and (F) show the distribution of the marker gene expressions in the RDEB cells and WT cells combined from the six pairs.

We also identified several genes as markers not previously associated with RDEB. These included *COL11A1*, a minor fibrillar collagen shown to mark activated cancer-associated fibroblasts (CAFs) that is not typically expressed in fibroblasts associated with inflammation and fibrosis [37]. scVDMC also revealed *GREM1*, a BMP antagonist associated with renal and pancreatic fibrosis [38, 39] and *MFAP5*, which promotes attachment of cells to micro-fibrils of the extracellular matrix and interacts with TGB*β* growth factors [40]. We performed flow cytometry on the same RDEB patient and matched sibling fibroblasts to validate the expression levels of these genes at the single-cell level and found the results similar to our RNA expression data shown in Fig 7. To further investigate the expressions of the these markers among the cells in the six populations, we plot the distribution of the cells with highly expressed markers in the six pairs in Fig 8. In the plots, the expression patterns of *GREM1* and *MFAP5* are very consistent among the cells in all the six pairs with more enrichment in RDEB cells (*GREM1*) or WT cells (*MFAP5*). The expression pattern of *COL11A1* is consistent in five of the pairs with enrichment in WT cells except RDEB-WT pair 3. Since the markers are selected to capture cell types rather than RDEB vs WT, there might be some discrepancy in the expression patters in each individual cell populations depending on the proportion of the cell types. As top hits, these genes potentially mark sub-populations of stromal cells that contribute to the transformation of the overlying epithelium and the development of squamous cell carcinoma in RDEB patients.

## Discussion

In this research, we demonstrated multitask learning is useful in analysis across multiple single-cell populations. It is also possible to apply other multitask learning or transfer learning methods [41] for the clustering tasks. scVDMC is a multitask clustering method specifically designed for scRNA-seq data for selection of a smaller set of cell-type markers and allows large variability in gene expression across the cell populations. Other methods are often built using different assumptions of the data that might not be applicable to the characteristics of scRNA-seq populations [42–44].

The amount of variation across multiple scRNA-Seq datasets depends on the nature of the datasets for the integrative analysis. For example, while we expect little variances among technical replicates and slightly more variances among biological replicates such that the variances do not play a major role in the pooled analysis, much larger variances might exist among samples of different tissue types or samples from different patients as those in the RDEB data. The key hypothesis of scVDMC is the existence of a common set of a small number of marker genes in every dataset that can partition each dataset into the same clusters. While the hypothesis is quite independent of the amount of variation across the datasets, scVDMC formulation accounts for the variation by tuning the parameter *α* to weight the variances. In theory, scVDMC is applicable to the general integration of scRNA-Seq datasets if the variances calculated among the cluster centers across the datasets well represent the underlying variations. However, in real applications, it is difficult to assess if the variations are captured by the computation of the variances. Thus, more careful practice of parameter tuning and validation of the results are necessary after the application of scVDMC.

There are limitations in the scVDMC method. In multitask clustering, assuming a global *k* as the number of clusters in each cell population dataset does not always hold true as for some rare cell types, the corresponding cells may only be present in some populations. scVDMC might incorrectly split a cluster of one cell type because no empty cluster is allowed. One possible improvement is to model each domain with an individual *k*^(*d*)^ with a more adaptive strategy for choosing *k*^(*d*)^. In this case, the overall balance between within-cluster distance and the variance will need to be more carefully weighted. In addition, cell-cycle-associated genes could be a large source of confounders. Unless the stages of cell cycle are the biological signal under study, cell cycle-related variation could obscure biological signals of interest. It is possible to model the confounders directly in the scVDMC method with more complex modeling. Alternatively, we could pre-process the scRNA-seq data to remove the cell cycle signals. For example, a Gaussian processes-based latent-variable model [45] was used to account for confounding variations due to the cell cycle in scRNA-seq data sets and then linear regression was applied to remove them. In this approach, a clearly defined cell cycle gene set is necessary to avoid removing true signals unexpectedly. Combined with the pre-precessing, scVDMC might achieve further improvement in clustering multiple cell populations.

For a better interpretation of scRNA-seq data, CellTree [17] based on Latent Dirichlet allocation also provides soft cluster assignment as opposed to the hard one-cluster assignment and more recently, a new method [46] was introduced for visualizing the cluster membership of single cells by the soft cluster assignment known as “grades of membership”. It is also possible to extend scVDMC method to perform soft cluster assignment by relaxing *V* to contain positive real numbers rather than binary 0/1 in Eq 2. The relaxation will require solving many least-squares problems and increase the computational time complexity. We plan to investigate better solutions of scVDMC in the future for soft cluster assignment and handling cell-cycle-associated gene signatures.

## Supporting information

S1 Appendix Minimizing Eq 3. (PDF)

S1 Fig scVDMC clustering results under varying *w* on the mESC data and Lung data. (TIF)

S2 Fig Convergence of scVDMC. The object function in Eq 1 is plotted under each iteration on the four datasets. In (A), (B) and (C), the parameters are *α* = 1, *W* = 0.1 and λ = 50. In (D), the parameters are *α* = 1, *W* = 0.5 and λ = 300, and the number of samples used is 1000 from donor A.
(TIF)

S3 Fig Read counts in the single cells. The total number of the reads and the number of aligned reads are shown in each single-cell library. RDEB and WT individual pairs are indicated below.
(TIF)

S4 Fig Capturing distinct single-cell populations by tuning *α*. PCA is applied to the single cell profiles of the marker genes learned by scVMDC from the combined cell populations in each dataset. Each plot shows the projection of the data and the cluster centers by the first two principle components. The clusters are shown in different colors and the cluster centers are indicated by the diamonds. The projections with *α* = 0 and 1 are compared on LUNG and mESC data and the projection with *α* = 0 and 5 are compared on PBMC data. In (E) and (F), the data and the cluster centers are shown seperately.
(TIF)

S5 Fig Pooled clustering of RDEB data with SC3. SC3 was applied to cluster the single-cell populations from the six RDEB-WT pairs. PCA was applied to project the combined single cell profiles of all the genes from the pooled six cell populations in the first three PCs.
(TIF)

S6 Fig Determining the number of clusters in PBMC data with “elbow” plot. The mean total within-clusters sum of squares of the clustering averaged in ten repeats are shown for different choices of the number of clusters. The optimal number of clusters is around 10 in all the three donors.
(TIF)

S7 Fig Determining the number of clusters in RDEB data with “elbow” plot. The mean total within-clusters sum of squares of the clustering averaged in ten repeats are shown for different choices of the number of clusters. The “elbow” starts from 4 in all the six RDEB-WT pairs.
(TIF)

S1 Table RDEB patient and donor demographics. RDEB patient and HLA-matched sibling age and gender at the time of sample collection.
(XLSX)

S2 Table Primary antibodies for flow cytometry. (XLSX)

S3 Table Secondary antibodies used for flow cytometry. (XLSX)
