# Supplementary material for: A multitask clustering approach for single-cell RNA-seq analysis in Recessive Dystrophic Epidermolysis Bullosa
Source: PLoS Comput Biol. 2018 Apr 9;14(4):e1006053. doi: 10.1371/journal.pcbi.1006053 (PMC5908193; doi:10.1371/journal.pcbi.1006053)
Supplement: S1 Appendix — (PDF) [file pcbi.1006053.s001.pdf]

S1 Appendix. Minimizing Equation 3: For simplicity, let's replace objective function in equation (3) by

$$\mathcal{J}(U^{(d)}) = \frac{1}{2}\mathcal{J}_1 - w\mathcal{J}_2 + \alpha\mathcal{J}_3. \tag{10}$$

Then the gradient of $U_{i,:}^{(d)T}$ is obtained as

$$\frac{\partial \mathcal{J}}{\partial(U_{i,:}^{(d)T})} = \frac{1}{2}\frac{\partial \mathcal{J}_1}{\partial(U_{i,:}^{(d)T})} - w\frac{\partial \mathcal{J}_2}{\partial(U_{i,:}^{(d)T})} + \alpha\frac{\partial \mathcal{J}_3}{\partial(U_{i,:}^{(d)T})}. \tag{11}$$

Let $\Psi = \mathbf{I}_k - \frac{\mathbf{1}_k\mathbf{1}_k^T}{k}$ and $\Phi = \mathbf{I}_d - \frac{\mathbf{1}_d\mathbf{1}_d^T}{d}$, we have

$$\frac{\partial \mathcal{J}_1}{\partial(U_{i,:}^{(d)T})} = 2B_i V^{(d)T}(V^{(d)}U_{i,:}^{(d)T} - X_{i,:}^{(d)T}) \tag{12}$$

$$\frac{\partial \mathcal{J}_2}{\partial(U_{i,:}^{(d)T})} = \frac{2B_i}{k}\Psi U_{i,:}^{(d)T} \tag{13}$$

$$\frac{\partial \mathcal{J}_3}{\partial(U_{i,:}^{(d)T})} = \frac{2B_i k}{d}(\Phi_{d,d}U_{i,:}^{(d)T} + \sum_{l\neq d}\Phi_{dl}U_{i,:}^{(l)T}). \tag{14}$$

Finally, bringing equations (12), (13) and (14) back into (11) we have

$$\begin{aligned}\frac{\partial \mathcal{J}}{\partial(U_{i,:}^{(d)T})} &= B_i(V^{(d)T}V^{(d)} - \frac{2w}{k}\Psi + \frac{2\alpha k\Phi_{d,d}}{d}\mathbf{I}_k)U_{i,:}^{(d)T} \\ &- B_i V^{(d)T}X_{i,:}^{(d)T} + \frac{2B_i\alpha k}{d}\sum_{l\neq d}\Phi_{dl}U_{i,:}^{(l)T}.\end{aligned} \tag{15}$$

When w and α are properly chosen (see section) and $B_i = 1$, objective (10) is convex. By setting the derivative (15) to zero we have the analytical solution

$$\begin{aligned}U_{i,:}^{(d)^T} &= (V^{(d)^T}V^{(d)} - \frac{2w}{k}\Psi + \frac{2\alpha k\Phi_{d,d}}{d}\mathbf{I}_k)^{-1}(V^{(d)^T}X_{i,:}^{(d)^T} - \\ &\frac{2\alpha k}{d}\sum_{l\neq d}\Phi_{dl}U_{i,:}^{(l)T}).\end{aligned} \tag{16}$$
