# Supplementary figures and images for: A multitask clustering approach for single-cell RNA-seq analysis in Recessive Dystrophic Epidermolysis Bullosa
Source: PLoS Comput Biol. 2018 Apr 9;14(4):e1006053. doi: 10.1371/journal.pcbi.1006053 (PMC5908193; doi:10.1371/journal.pcbi.1006053)

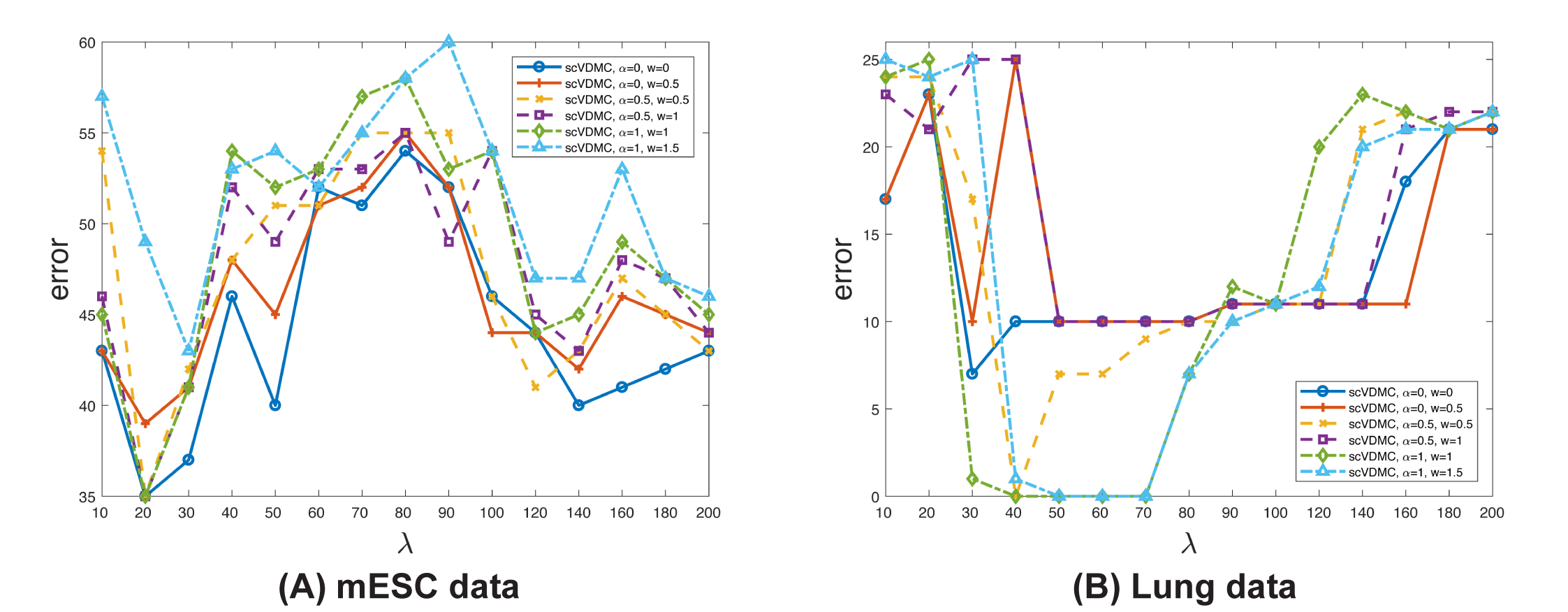

Supplement: S1 Fig — (TIF) [file pcbi.1006053.s002.tif]

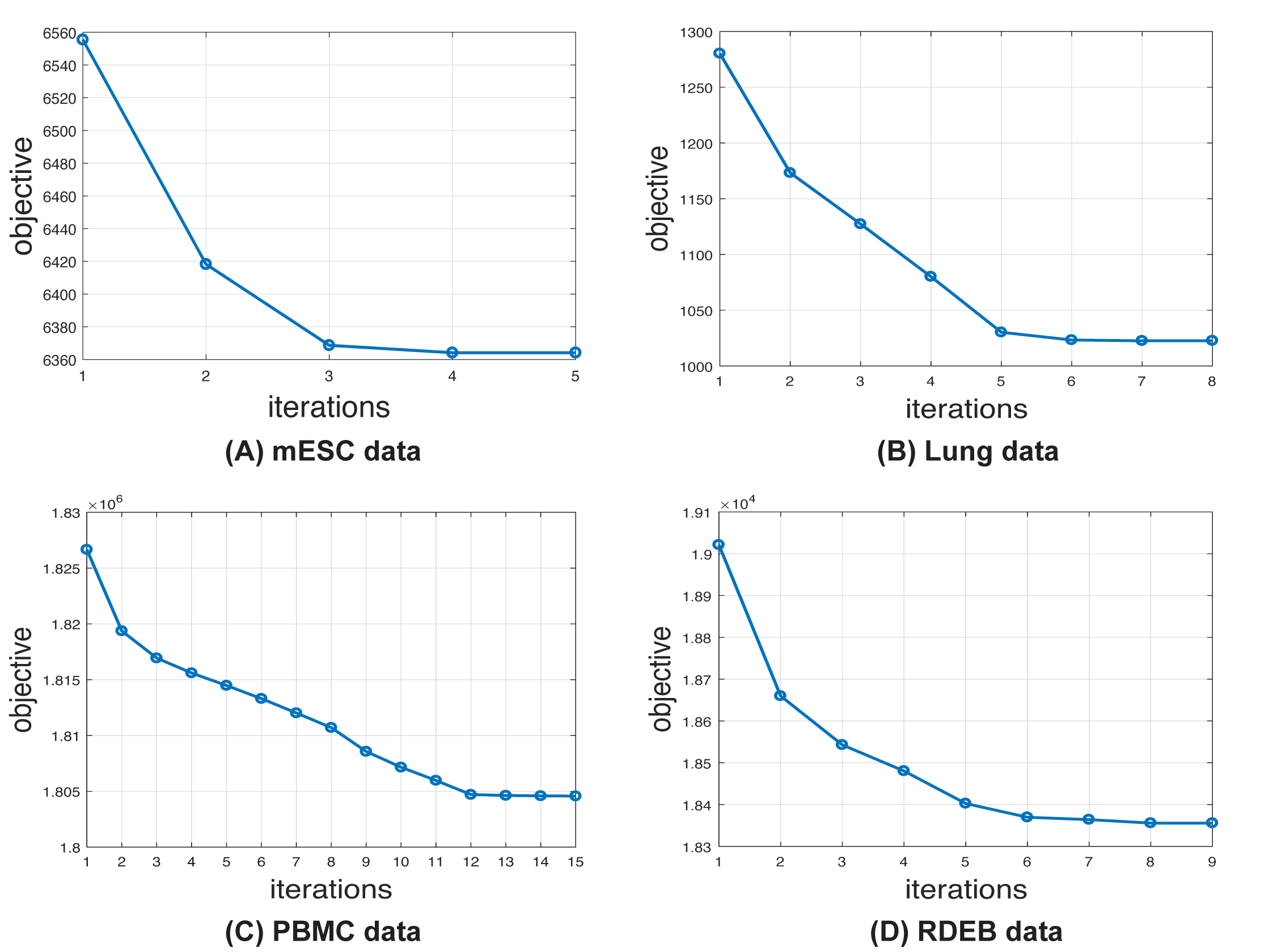

Supplement: S2 Fig — The object function in Eq 1 is plotted under each iteration on the four datasets. In (A), (B) and (C), the parameters are α = 1, W = 0.1 and λ = 50. In (D), the parameters are α = 1, W = 0.5 and λ = 300, and the number of samples used is 1000 from donor A. (TIF) [file pcbi.1006053.s003.tif]

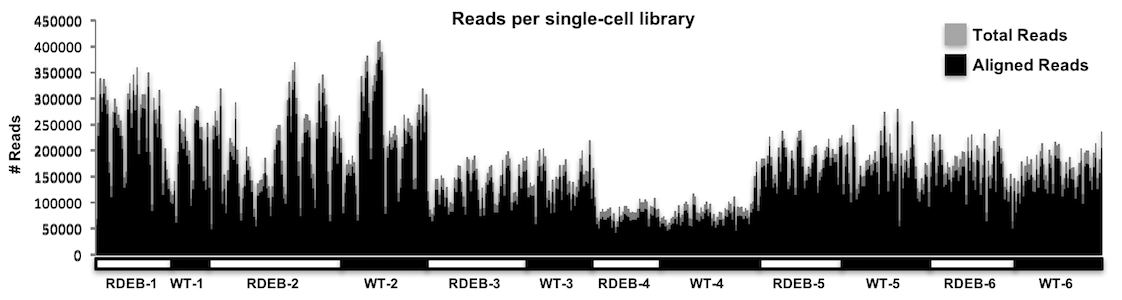

Supplement: S3 Fig — The total number of the reads and the number of aligned reads are shown in each single-cell library. RDEB and WT individual pairs are indicated below. (TIF) [file pcbi.1006053.s004.tif]

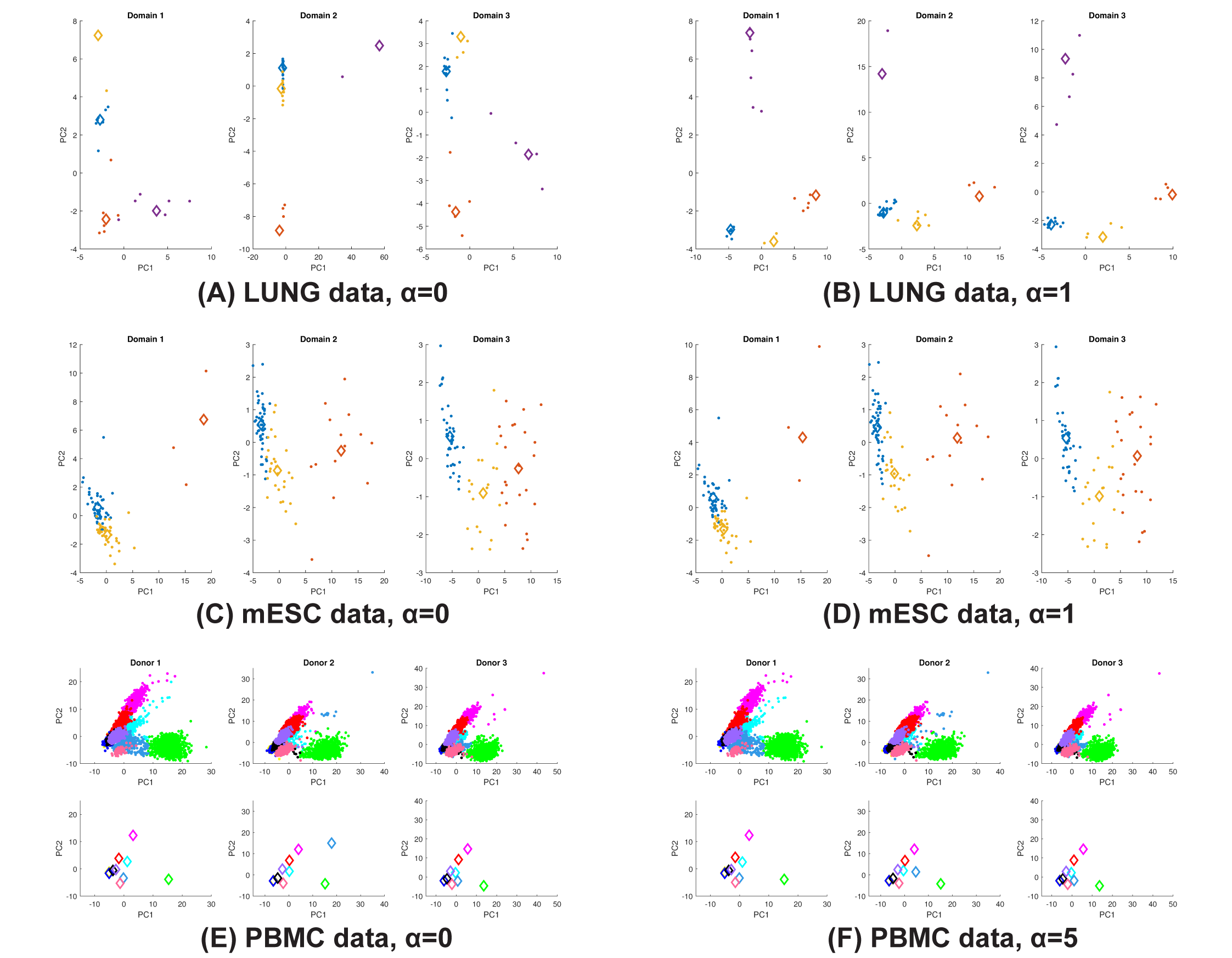

Supplement: S4 Fig — PCA is applied to the single cell profiles of the marker genes learned by scVMDC from the combined cell populations in each dataset. Each plot shows the projection of the data and the cluster centers by the first two principle components. The clusters are shown in different colors and the cluster centers are indicated by the diamonds. The projections with α = 0 and 1 are compared on LUNG and mESC data and the projection with α = 0 and 5 are compared on PBMC data. In (E) and (F), the data and the cluster centers are shown seperately. (TIF) [file pcbi.1006053.s005.tif]

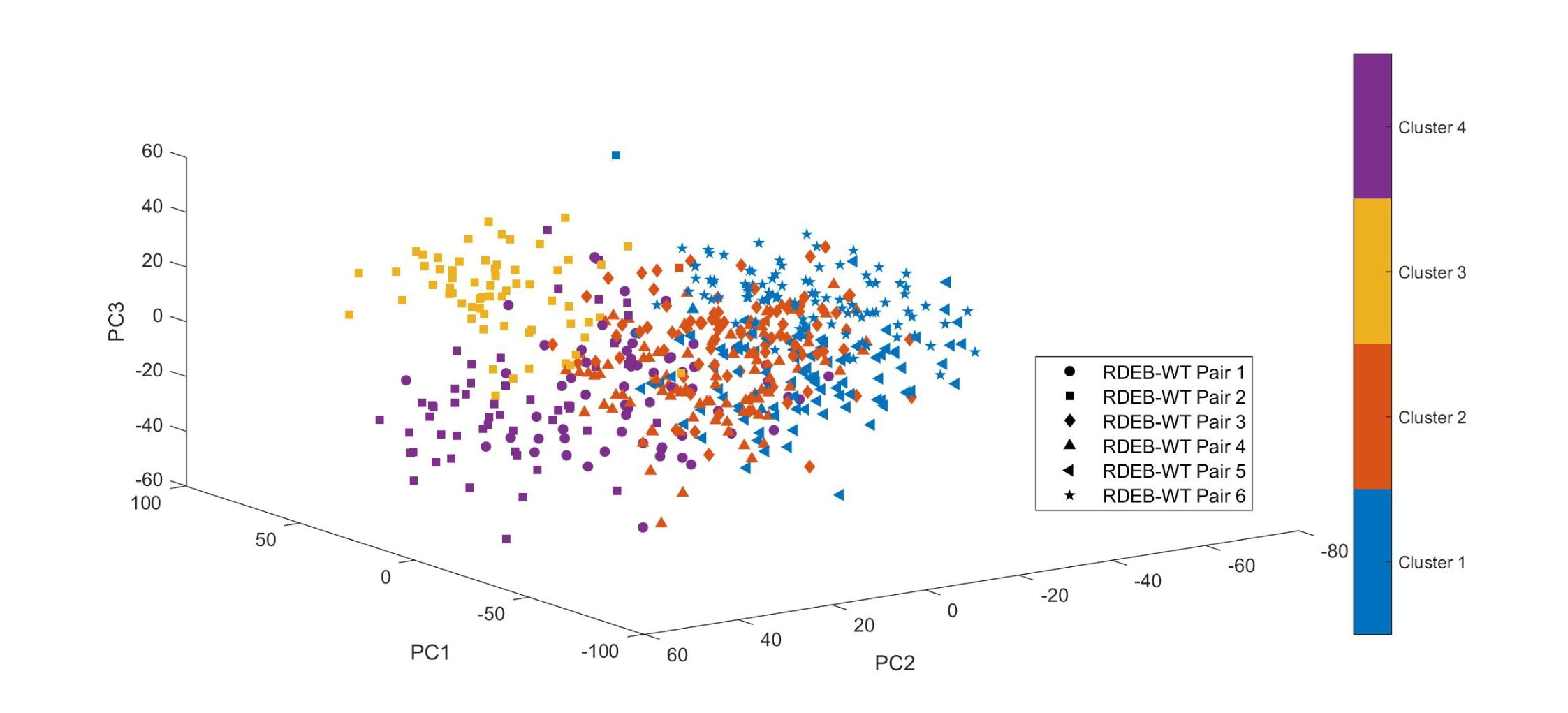

Supplement: S5 Fig — SC3 was applied to cluster the single-cell populations from the six RDEB-WT pairs. PCA was applied to project the combined single cell profiles of all the genes from the pooled six cell populations in the first three PCs. (TIF) [file pcbi.1006053.s006.tif]

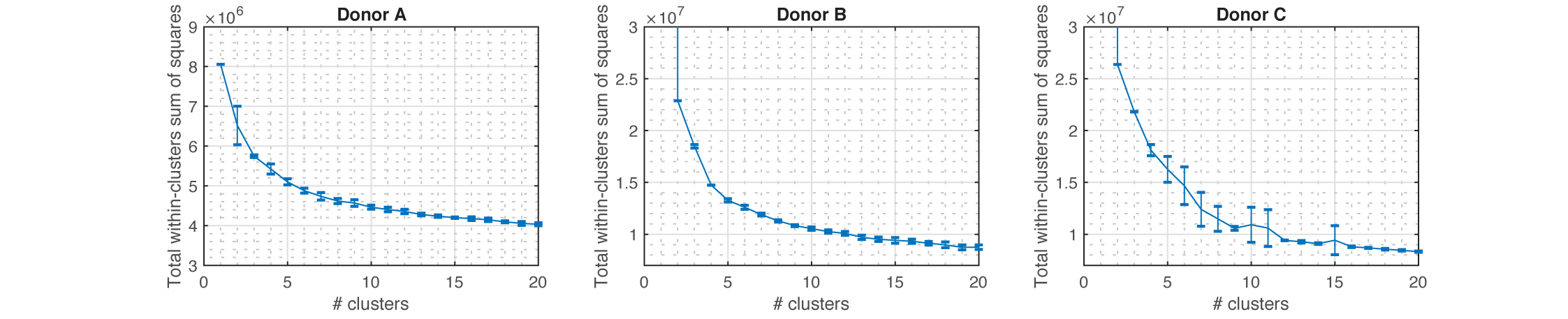

Supplement: S6 Fig — The mean total within-clusters sum of squares of the clustering averaged in ten repeats are shown for different choices of the number of clusters. The optimal number of clusters is around 10 in all the three donors. (TIF) [file pcbi.1006053.s007.tif]

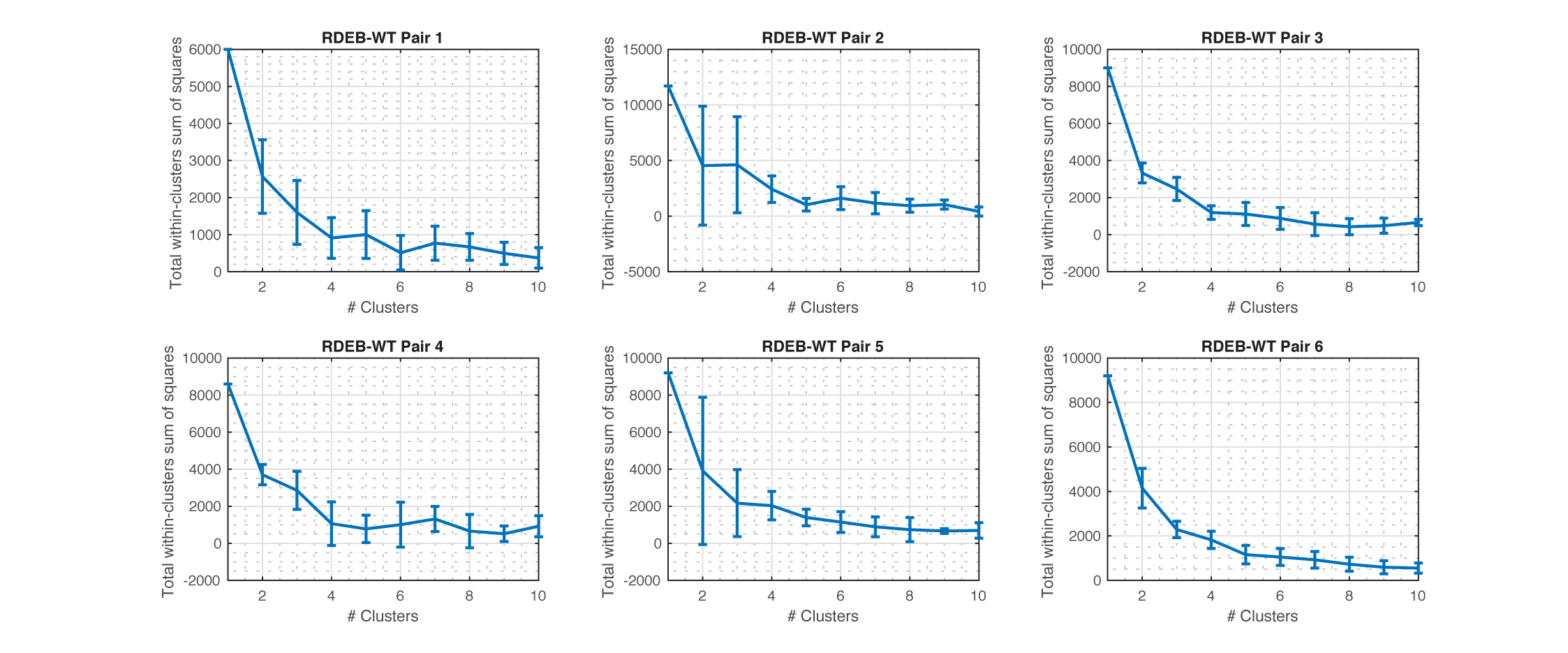

Supplement: S7 Fig — The mean total within-clusters sum of squares of the clustering averaged in ten repeats are shown for different choices of the number of clusters. The “elbow” starts from 4 in all the six RDEB-WT pairs. (TIF) [file pcbi.1006053.s008.tif]
